# Intrinsic Formamidinium Tin Iodide Nanocrystals by Suppressing the Sn(IV) Impurities

**DOI:** 10.1021/acs.nanolett.2c04927

**Published:** 2023-02-28

**Authors:** Dmitry N. Dirin, Anna Vivani, Marios Zacharias, Taras V. Sekh, Ihor Cherniukh, Sergii Yakunin, Federica Bertolotti, Marcel Aebli, Richard D. Schaller, Alexander Wieczorek, Sebastian Siol, Claudia Cancellieri, Lars P. H. Jeurgens, Norberto Masciocchi, Antonietta Guagliardi, Laurent Pedesseau, Jacky Even, Maksym V. Kovalenko, Maryna I. Bodnarchuk

**Affiliations:** † Institute of Inorganic Chemistry, Department of Chemistry and Applied Biosciences, ETH Zürich, CH-8093 Zürich, Switzerland; ‡ Empa−Swiss Federal Laboratories for Materials Science and Technology, CH-8600 Dübendorf, Switzerland; § Dipartimento di Scienza e Alta Tecnologia & To.Sca.Lab, Università dell’Insubria, 22100 Como, Italy; ∥ Univ Rennes, INSA Rennes, CNRS, Institut FOTON, Rennes F-35000, France; ⊥ Center for Nanoscale Materials, Argonne National Laboratory, Lemont, Illinois 60439, United States; # Department of Chemistry, Northwestern University, Evanston, Illinois 60208, United States; ∇ Istituto di Cristallografia & To.Sca.Lab, Consiglio Nazionale delle Ricerche, 22100 Como, Italy

**Keywords:** halide perovskite, lead-free, nanocrystals

## Abstract

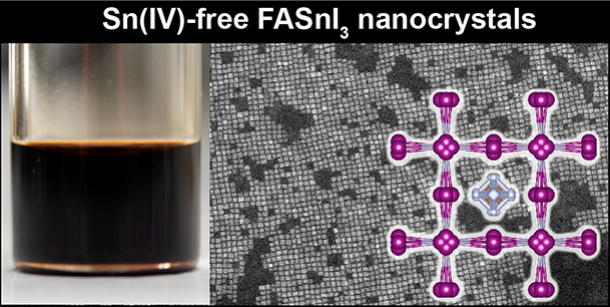

The long search for nontoxic alternatives to lead halide perovskites (LHPs) has shown that some compelling properties of LHPs, such as low effective masses of carriers, can only be attained in their closest Sn(II) and Ge(II) analogues, despite their tendency toward oxidation. Judicious choice of chemistry allowed formamidinium tin iodide (FASnI_3_) to reach a power conversion efficiency of 14.81% in photovoltaic devices. This progress motivated us to develop a synthesis of colloidal FASnI_3_ NCs with a concentration of Sn(IV) reduced to an insignificant level and to probe their intrinsic structural and optical properties. Intrinsic FASnI_3_ NCs exhibit unusually low absorption coefficients of 4 × 10^3^ cm^–1^ at the first excitonic transition, a 190 meV increase of the band gap as compared to the bulk material, and a lack of excitonic resonances. These features are attributed to a highly disordered lattice, distinct from the bulk FASnI_3_ as supported by structural characterizations and first-principles calculations.

In a decade since the landmark works of the Snaith and Grätzel groups,^1,2^ lead halide perovskites (LHPs) have paved their way aside from photovoltaics toward many eminently different applications ranging from backlit displays and light-emitting diodes to hard radiation and neutron detection.^3−15^ Such a broad utility of LHPs is rooted in a rare combination of their properties, including low density of carrier trap states (10^9^–10^10^ cm^–3^) despite a large density of point defects,^5,16,17^ long electron–hole diffusion lengths (2–175 μm),^5,18,19^ high carrier mobilities (2.5–1000 cm^2^ V^–1^ s^–1^),^5,17,20−22^ long charge carrier lifetimes (0.08–450 μs),^5,18−21,23^ small carrier effective masses (0.069–0.25 m_0_),^24^ and high optical absorption coefficients at the absorption edge (2–7 × 10^4^ cm^–1^).^17^ Although LHP-based devices may meet RoHS compliancy in some cases,^25,26^ the anticipated scale of the LHP market calls for lead-free alternatives.^27^

Compelling electronic characteristics of LHPs largely arise from the pronounced tolerance to intrinsic defects^28,29^ and lattice softness allowing large polarons and efficient screening of the carriers.^30−33^ Tin(II)- and germanium(II)-based AMX_3_ halide perovskites [A = Cs, formamidinium (FA), or methylammonium (MA)] have always been envisioned as the closest alternatives to LHPs. The metal–halide–metal (M–X–M) angle of about 180° ensures the highest σ overlap of the orbitals and the efficient dispersion of the resulting electronic bands. Tilting the octahedra or alternating cations in other halide perovskitoids reduces orbital overlap, opens the band gap, and leads to heavier carriers.^34,35^ Metal halides with edge- or face-sharing octahedra, or even with fully isolated ones (0D-halides), exhibit properties that are notably different from the prototypic LHPs. Although these properties are promising for some applications,^36−47^ such materials are unlikely to deliver the photovoltaic performance or narrowband excitonic luminescence similar to LHPs.

The main factor limiting the performance of Sn and Ge halide perovskites is their low stability toward oxidation, which by a number of pathways can lead to degenerate p-type conductivity.^48^ Nevertheless, considerable progress has been achieved in FASnI_3_-based photovoltaics over the past seven years with a 7-fold increase of the power conversion efficiency (PCE), with a benchmark now of 14.81% (Figure S1).^49,50^ Synthetic approaches can be grouped as follows:

(i) reduction of the present Sn(IV) impurities by comproportionation with metallic Sn(0)^51,52^
(ii) altering the lattice of FASnI_3_ by doping with bifunctional organic cations capable of pinning Sn vacancies (so-called “hollow” structures)^53−58^
(iii) passivation of FASnI_3_ surface with bulky organic cations^59^

In contrast, the synthesis of tin halide perovskite nanocrystals (NCs) remains scarce in the literature and is mainly limited to CsSnX_3_ or mixed APb_*y*_Sn_1–*y*_X_3_ compositions.^61−69^ L. Dai et al. have recently reported the protocol allowing the synthesis of FASnI_3_ NCs with good morphological quality.^70^ However, their work focuses on the hot-carrier relaxation processes in FASnI_3_ NCs and does not discuss the effect of the present impurities on the optical properties of NCs, which we find to be crucial.

We thus sought to develop a colloidal synthesis of FASnI_3_ NCs with high morphological quality and purity and study their intrinsic optical properties and structure, as reported herein. We devote special care to the purity of all of the involved precursors to exclude possible doping during the synthesis. We show that pure FASnI_3_ NCs exhibit a disorder of the I-sites that reduces the Sn–I–Sn angle to 167° and opens the band gap by ∼190 meV. Our findings are supported by ab initio calculations for the average cubic structure of FASnI_3_ showing that the octahedra exhibit a significant tilting that profoundly affects the electronic structure. Such distortion also allows for unusually pronounced photoinduced states. Based on these findings, we propose an additional pathway for tuning the optical properties of FASnI_3_ NCs via altering their lattice with bifunctional organic cations.

In the footstep of our earlier reports on the synthesis of FAPbI_3_ NCs, the first edition of FASnI_3_ NCs involved 1-octadecene (ODE) as a solvent (Figure S2). Briefly, oleylamine (OAm), oleic acid (OA), and formamidinium oleate are sequentially injected into a hot SnI_2_ solution in trioctylphosphine (TOP) and ODE. However, we have found two rarely considered important synthetic parameters to be crucial for the synthesis of Sn(IV)-free FASnI_3_ NCs (see Supporting Information Note S1 for details). First, all tested commercially available sources of SnI_2_ are of >99% metal basis purity but contain significant amounts of SnO, SnO_2_, and SnI_4_ that notably affect the synthesis. Therefore, we have developed a two-step purification procedure that results in SnI_2_ with an insignificant amount of Sn(IV) species, as evidenced by X-ray photoelectron spectroscopy (XPS) and X-ray diffraction (XRD). Second, reactions performed at 80 °C in ODE nearly instantly lead to the oxidation of iodide anions to polyiodide ones, apparent from their characteristic red-brown color. Note that photoluminescence (PL) spectra of NCs synthesized in ODE do not correlate with their size, and the PL maximum fluctuates in a broad range from 700 to 850 nm (Figure S3). Therefore, we opt for a synthesis in aromatic solvents (toluene, mesitylene, or Dowtherm A) that proceeds without color change until formamidinium oleate solution is injected and NCs start to grow (Figures S4 and S5).

The optimized synthesis proceeds by solubilizing SnI_2_ in Dowtherm A in the presence of oleylamine and TOP, followed by the hot injection of formamidinium oleate dissolved in Dowtherm A (see the SI for the details). The stability of as-prepared NCs is sufficient to wash them by precipitation with acetonitrile, if washing is performed air-free. This protocol allows the removal of all byproducts and impurities of layered perovskites with PL around 700 nm, which is always present in the crude solution (Figures 1a–c).

**Figure 1:**
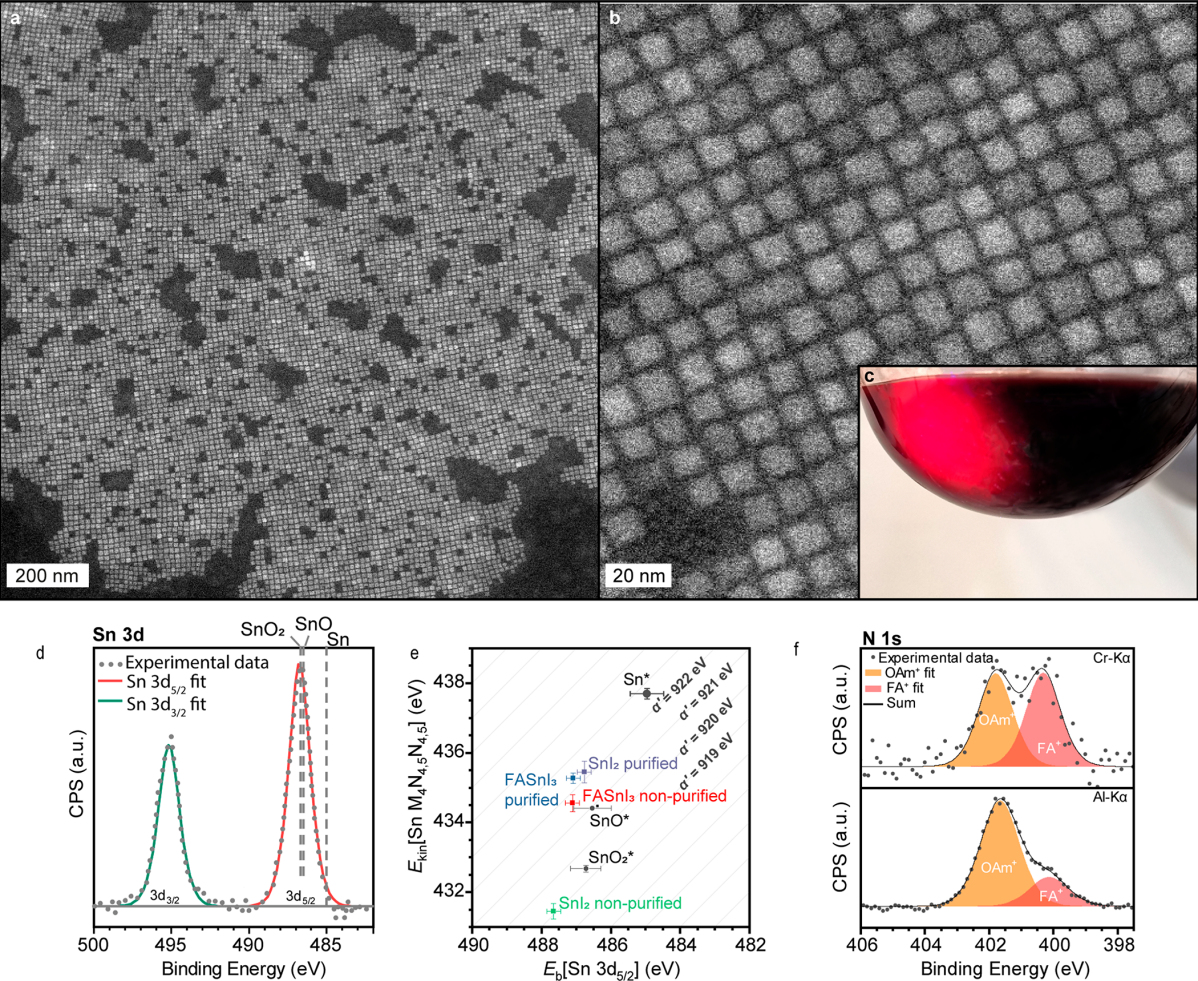
(a, b) DF STEM images of representative FASnI_3_ NCs. (c) Visual appearance of the crude solution of FASnI_3_ NCs. (d) XPS Sn 3d spectra of FASnI_3_ NCs synthesized from purified SnI_2_ with corresponding fits and reference peaks for Sn, SnO, and SnO_2_ extracted from the NIST database.^60^ (e) Wagner plot illustrating modified Auger parameters of Sn in FASnI_3_ NCs made from purified and nonpurified SnI_2_ with the respective references for Sn, SnI_2_, SnO, and SnO_2_. (f) HAXPES N 1s spectra made with hard (Cr Kα) and soft (Al Kα) X-ray sources.

XPS combined with inert-gas transfer was used to collect the Sn 3d core level spectra.^71^ The acquired spectra for Sn 3d_5/2_ and 3d_3/2_ do not show apparent asymmetry and can be well-fitted with a single component, indicating Sn is mainly present in one oxidation state (Figure 1d). FASnI_3_ samples made from purified and nonpurified SnI_2_ showed great similarity (Figure S6). However, due to the high proximity of peaks corresponding to Sn^2+^ and Sn^4+^ and inaccuracies related to the referencing of the binding energy scale as well as charging effects, the spectrum analysis can sometimes be ambiguous, leading to possibly wrong assumptions about the oxidation state. This is especially the case with low Sn^2+^ or Sn^4+^ contents. The modified Auger parameter, *α′* (AP),^72^ allows one to cancel out shifts related to charging and band bending effects and was shown to be useful for resolving the oxidation state and chemical environment of Sn in perovskite-type structures, including polycrystalline FASnI_3_ films.^71^ The value of *α′* corresponds to the sum of the binding energy (BE) of the Sn 3d^5^/_2_ photoelectron line and the kinetic energy (KE) of the corresponding Sn M_4_N_4,5_N_4,5_ Auger line. Any shift in *α′* can be directly related to a change in the local electronic polarizability,^72^ which is extremely sensitive to changes in the local chemical state of the atom in the compound.^73,74^ Shifts in *α′* can be visualized in a so-called chemical-state (or Wagner) plot, where constant *α′* values lie on diagonal lines (Figure 1e). Strikingly, the *α′* value for nonpurified SnI_2_ (919.1 eV) is highly similar to that reported for SnO_2_, indicating its presence and potentially that of other Sn(IV) compounds on the powder surface. The purified SnI_2_, in contrast, exhibits a higher *α′* value (922.2 eV), similar to other Sn(II) compounds. Likewise, the *α′* values of the FASnI_3_ NC films made from purified and nonpurified SnI_2_ significantly differ (922.3 eV vs 921.6 eV), with the first value matching precisely the one reported for polycrystalline FASnI_3_ films.^71^ These data demonstrate the enhanced sensitivity of the AP analysis for detecting minuscule differences in the local chemical state of Sn. In agreement with XPS data, we have not observed any signs of the oxidized FA_2_SnI_6_ form in ^119^Sn solid-state NMR, which is expected at −4818 ppm.^75^ We also did not observe the effect of a number of reducing agents on the optical properties of FASnI_3_ NCs, as summarized in Supporting Information Note S2. These findings suggest that the amount of Sn(IV) impurities in the synthesized FASnI_3_ NCs is insignificant and should not affect the optical properties of the obtained NCs.

The core region of the FASnI_3_ NCs can be more effectively probed by hard X-ray photoelectron spectroscopy (HAXPES) analysis using a hard Cr Kα X-ray source, resulting in an approximate increase of the information depth by a factor of 3.^76^ This comparison helps us to study the distribution of N in the NC core and on the surface in the form of formamidinium (N_FA_^+^) and oleylammonium (N_OAm_^+^), respectively (Figure 1f). HAXPES reveals that the total N_OAm_^+^/N_FA_^+^ ratio (1:1) is lowered compared to that of the XPS analysis (3.6:1), which probes the surface of NCs preferentially. This finding indicates that NCs in solution have a significant amount of unbound OAmI ligands, in agreement with the analogous CsPbBr_3_ NCs and ICP-MS data showing a strong excess of I over Sn.^77^

Pure colloidal FASnI_3_ NCs exhibit absorption edge and PL in the range 770–830 nm with PL quantum yield (QY) of 0.1%. (Figure 2a). Time-resolved emission spectra (TRES) are found to be wavelength-independent, confirming the uniformity of the NCs (Figures 2b, Figure S7). PL decay is biexponential with 0.3 ns (90%) and 1.9 ns (10%) lifetimes, in agreement with reported values for bulk FASnI_3_.^78^ The excitonic absorption peak of pure FASnI_3_ NCs is unresolved, similar to the previously reported spectra of other tin halide perovskite NCs.^62,65^ We note that the absorption tail lasting to ∼800 nm belongs to NCs and not to the scattering, as ensured by filtering the solution through a 0.2 μm PTFE filter and evidenced by PL upon excitation at 635 nm (Figure S8).

**Figure 2:**
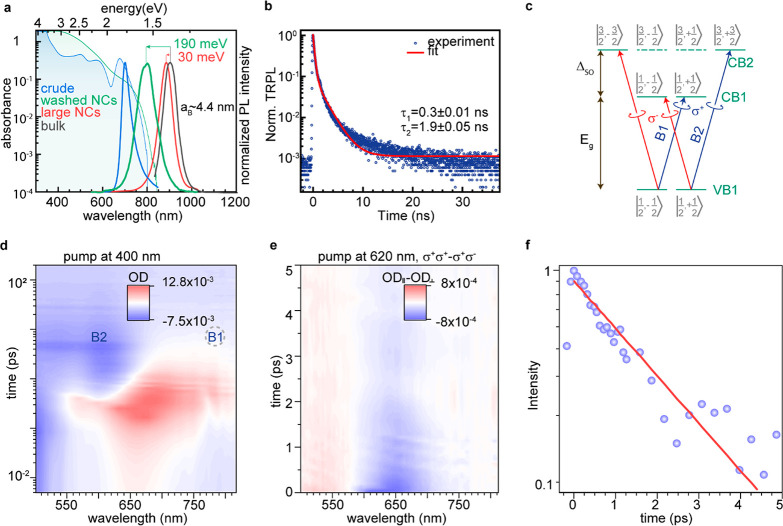
(a) Absorption and PL spectra of FASnI_3_ NCs; PL spectra of bulk FASnI_3_ and 200 nm large nanocrystals are shown for comparison. (b) Time-resolved PL spectrum of FASnI_3_ NCs in solution. (c) Optical selection rules in FASnI_3_ and corresponding absorption transitions; the spin-polarized states are labeled using |*J*, *m*_*J*_⟩. The solid and dashed lines of the *J* = 3/2 states represent heavy and light electrons correspondingly. (d) Pseudocolor 2D plot for room temperature pump–probe TA spectroscopy of colloidal FASnI_3_ NCs pumped at 400 nm. (e) Net spin pseudocolor 2D plot obtained as a difference between co- and cross-polarized TA spectra pumped at 620 nm. (f) Experimental (blue dots) net spin kinetics with 650 nm probe photons and single-exponent fit (red line).

Absorption edge and PL of the synthesized FASnI_3_ NCs are notably (by 190 meV) shifted from the band gap of bulk FASnI_3_ (Figure 2a). Although the increase of the band gap is expected for colloidal semiconductor NCs, such a big shift can hardly be explained solely by quantum confinement. For example, the confinement energy in FAPbI_3_ and CsPbI_3_ NCs of similar sizes is 89–93 meV.^17,79,80^ These NCs are about 10 nm large, whereas the exciton Bohr radius in FASnI_3_ should be 3.5–4.4 nm, depending on the set of effective masses reported for bulk material,^81,82^ indicating that these NCs should be in a weak confinement regime.

Absorption spectra of the pure FASnI_3_ NCs do not exhibit resolved excitonic transitions despite the narrow size dispersion (8–12%). Furthermore, these NCs exhibit rather low intrinsic absorption of about 4 ± 1.7 × 10^3^ cm^–1^ (determined 100 meV above the band gap by the ICP-MS with a combination of acidic and basic digestions for Sn and I, respectively; see Supporting Information Note S3 for details). That is about 4 times lower than the absorption coefficient reported for bulk FASnI_3_.^83^

Figure 2d shows spectrally resolved transient absorption (TA) data of FASnI_3_ NCs pumped at 400 nm, well above the band gap. This spectral map reveals a pronounced bleach around 600, ascribed to the VB1-CB2 transition (Figure 2c, see Supporting Information Note S4 for details).^84,85^ The net spin relaxation kinetics, evaluated as the difference between co- and cross-polarized TA signals, is fitted using a single-exponential decay and yields a lifetime of 2 ps, on par with the one of LHP NCs (∼1–3 ps), which is much shorter than in the case of CsSnBr_3_ NCs (Figures 2e,f).^85^

In addition to the expected bleaches, Figure 5d reveals a pronounced photoinduced absorption (PIA) band below the B2 bleach. Depending on the experimental conditions, this PIA band occurs on different time scales and at slightly different energies (Figure S9). In the case of resonance pumping, the PIA band is polarization-sensitive, appears at ∼0.2 ps, has a maximum at 703 nm, correlates with depopulation of B2, and can be explained as a biexciton shift.^76,85^ In the case of pumping at 400 nm, in contrast, PIA appears before the thermalization of carriers to the B2 state, has a maximum around 670 nm, and is also significantly more intense. This type of PIA is nearly absent with resonance pumping. We suggest that this PIA can arise due to the additional photoinduced states created upon lattice distortion induced by hot carriers. Fast PIA to these states becomes possible until the hot carrier is thermalized, and lattice distortion is released. Note that such strong PIA was not observed for CsPbX_3_.^76^ In contrast, electron localization has been recently predicted to be energetically favorable in many tin halide perovskites, with bipolaronic states being the most stable form of self-trapped electrons.^86^

We hypothesized that the unusually large band gap, distinct PIA, as well as weak and unresolved absorption transitions in FASnI_3_ NCs may be related to the reduced lattice symmetry, as evidenced below by wide-angle X-ray total scattering (WAXTS) and solid-state ^119^Sn NMR data.

FASnI_3_ NCs exhibit a cubic crystal structure and *Pm*3̅*m* space group symmetry (model parameters are reported in Table S1), as recently reported for FASnI_3_ bulk crystal (Figure 3a).^87,88^ Results from both Rietveld refinement^89^ and the Debye scattering equation (DSE)-based method^90^ agree on a disordered cubic model, where each iodine is replaced by four equivalent ions, offset by 0.36 Å from the original site and lying on a plane perpendicular to the direction of the Sn–Sn axis, each one with a fractional site occupancy factor (sof) of 0.25 (Figure 3b). Such a small local distortion makes the Sn–I–Sn angle bent (by about 13°), while the *Pm*3̅*m* cubic symmetry is retained. We verify this picture using density functional theory (DFT) calculations on the average cubic but disordered structure of FASnI_3_ that yield an average Sn–I–Sn bending of 10° (see computational details in the SI). The presence of this structural disorder is also suggested by the very large isotropic Debye–Waller factors (*B*) of iodide ions (*B*(I) > 6 Å^2^). Improved modeling of WAXTS data is achieved by splitting the iodide positions, which led to a better match of peak intensities (as detailed in the SI) and reduced *B*(I) values of 2.8 Å^2^. The same model has already been used to represent the structural disorder in FAPbI_3_ and FAPbBr_3_ NCs.^79,91^ Remarkably, although large atomic displacement parameters of iodine ions (6.3 Å^2^) are reported for bulk FASnI_3_,^87^ there was no indication of iodide displacement, while the archetypal cubic structure was favored (Table S2). Despite the considerably high *B*(Sn) factors (>4 Å^2^), WAXTS data for these NCs do not support the local disorder previously reported for bulk samples where Sn is off-centered along the [111] crystallographic direction^92^ (not observed in single crystal XRD data of ref (87); see Table S2).

**Figure 3:**
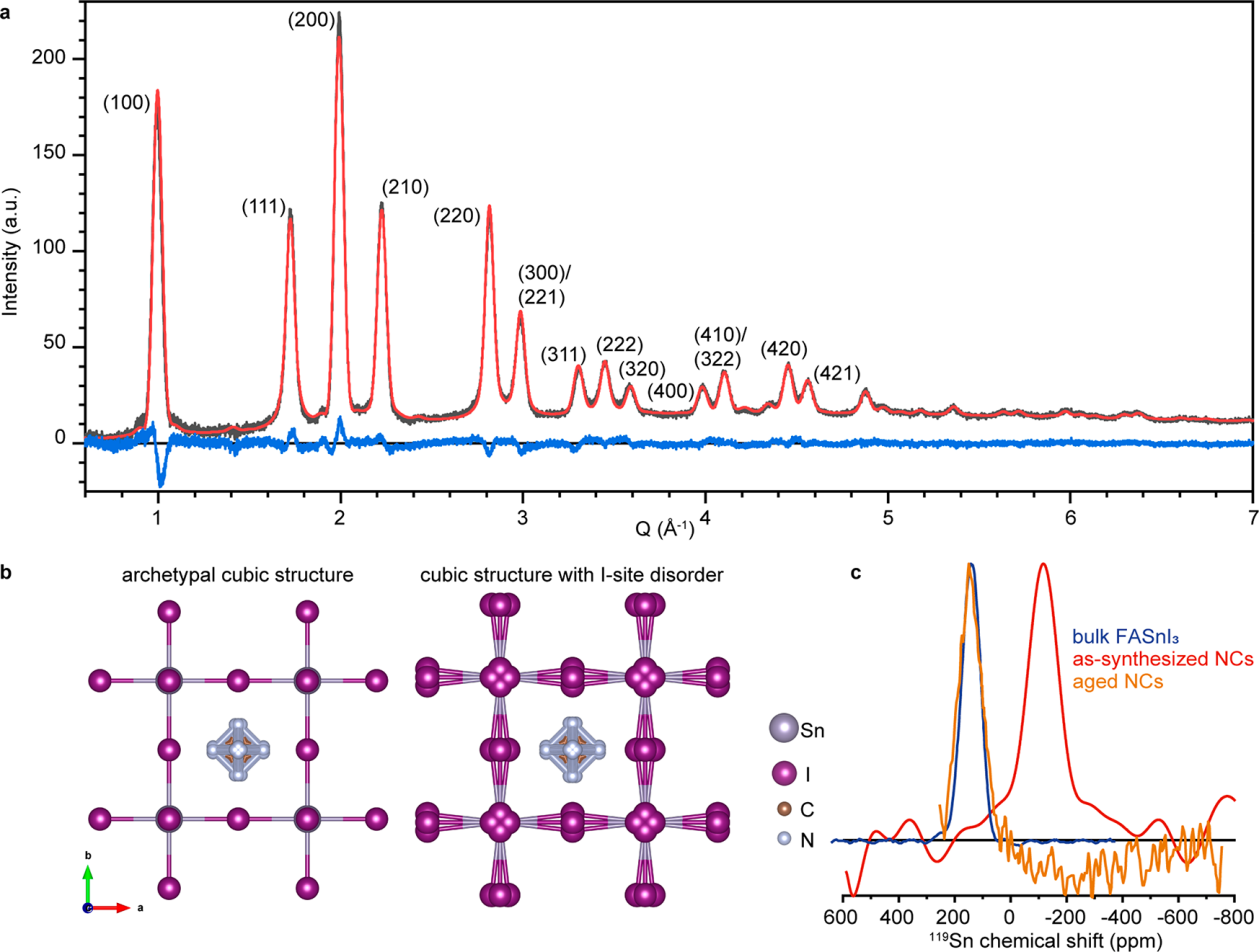
(a) Synchrotron WAXTS data (black curve) collected on colloidal solution of FASnI_3_ NCs plotted with the solvent signal subtracted, DSE simulation (red trace), and difference profile (blue curve) using the NC disordered cubic (*Pm*3̅*m* space group) model including (b) schematic of iodide displacement. In panel b, we also include the inorganic network with iodide disorder as calculated for DFT. (c) Solid-state ^119^Sn NMR spectra for bulk FASnI_3_, colloidal, and aged FASnI_3_ NCs.

Analogously to WAXTS data, ^119^Sn solid-state NMR indicates that the lattice symmetry of FASnI_3_ NCs might be reduced compared to bulk FASnI_3_: the chemical shift of FASnI_3_ NCs (−116 ppm) is significantly lower compared to bulk FASnI_3_ (140 ppm, Figure 3c). Note that, after aging and merging of NCs, the ^119^Sn chemical shift (144 ppm) coincides with the bulk material, indicating that the signal at −120 ppm is a signature of individual NCs that are not degraded.

Although the time-averaged structure of FASnI_3_ NCs is cubic, the observed type of disorder can increase the band gap. DFT calculations of the electron spectral function of the disordered cubic FASnI_3_ (color map) and the band structure of the archetypal cubic FASnI_3_ (black dots) show that the electronic structure is modified significantly upon allowing the system to accommodate local distortions (Figure 4). In particular, the band gap increases by ∼223 meV due to the reduced Sn–I–Sn angle. This phenomenon is well-known for metal halide perovskite-like compounds.^94^ The DOS around the band edges is consistent with the parabolic band approximation varying with the square root of energy. The peak structures in the conduction band reflect the nearly triply degenerate conduction band minimum at the R high symmetry point. Despite the presence of local distortions, the degeneracy should be maintained since the network reflects, on average, a high-symmetry structure. The DFT calculations also reveal the smearing of the electronic structure, which accounts for the absence of excitonic resonances in the absorption spectrum. Moreover, this smearing induces a difference between the Tauc plots for the “direct” or “indirect band model” (see Figure S10 and discussion therein), while still considering zero-phonon optical transition. These simulations show that the highly disordered average cubic lattice of FASnI_3_ may significantly depart from the standard picture of a perfectly ordered direct-band-gap semiconductor when optoelectronic properties are considered.

**Figure 4:**
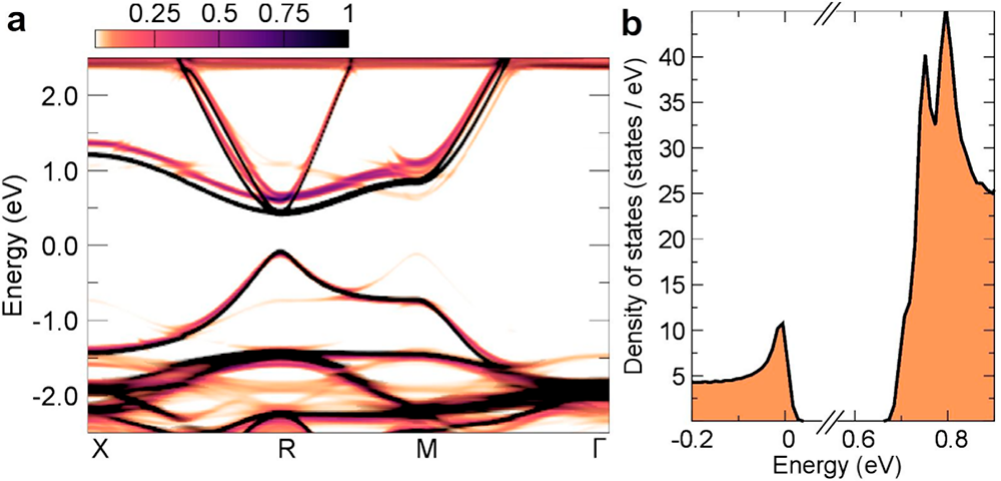
(a) Momentum-resolved electron spectral function and (b) density of states of FASnI_3_ calculated using the disordered structure in a 2 × 2 × 2 supercell and the band structure unfolding technique.^93^ Black lines in panel a represent the electron band structure calculated using the archetypal cubic FASnI_3_ structure at high-symmetry positions but with the FA molecules in orientations as calculated for the disordered structure.

The observed lattice disorder and PIA hint that intrinsic FASnI_3_ NCs are unlikely to exhibit optical properties resembling those of lead halide perovskite NCs. One way to reduce the remarkable, but in this case undesired, smearing of the electronic structure is to alter the lattice via mild doping. This motivated us to explore the possibility of tuning the optical properties of FASnI_3_ NCs by doping them with small A-site or bifunctional cations (Figure 5, Supporting Information Note S5). Indeed, doping with Cs or ethylenediammonium (up to 5%) leads to the onset of a slightly resolved absorption feature around 700 nm, which is absent in undoped FASnI_3_ NCs and smears away at higher levels of doping (Figure 5b). This trend coincides with a PL peak shift, notable narrowing, and PL QY dependence on the doping level (Figure 5c). The energy gap between the absorption band edge and PL peak position quickly decreases by 3 times (Figure 5c). Altogether, this indicates that doping may be the right strategy to overcome the observed disorder-induced smearing of the optoelectronic properties in the FASnI_3_ NCs.

**Figure 5:**
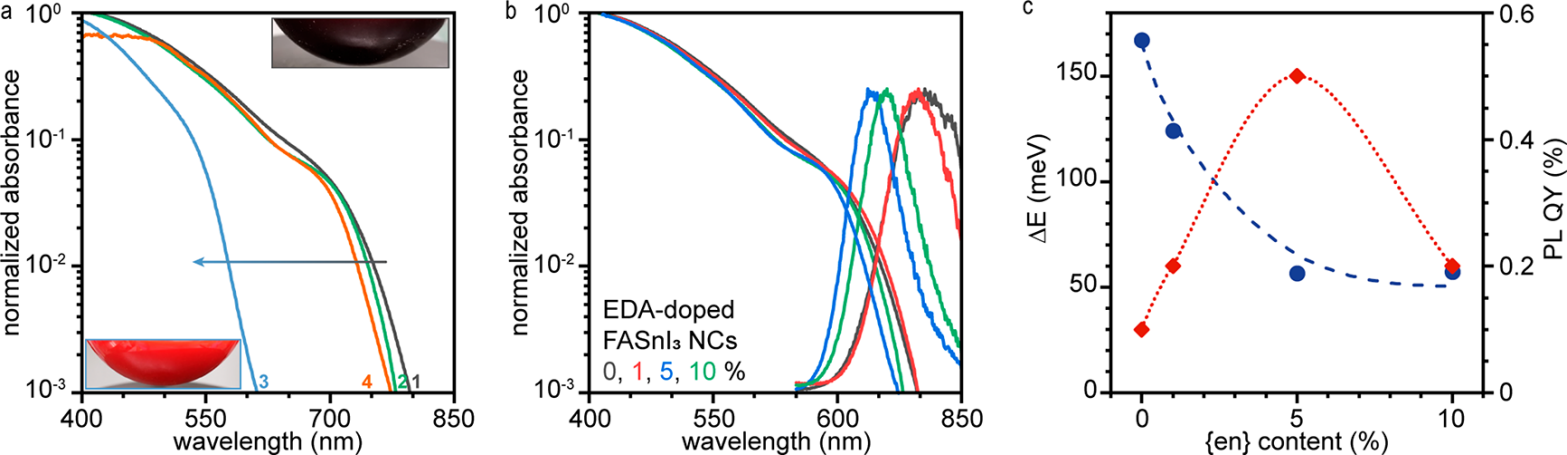
(a) Absorption spectra of undoped (1) colloidal FASnI_3_ NCs and NCs doped with hydroxyethylammonium (2), ethylenediammonium (3), and Cs (4). (b) Absorption and PL spectra of FASnI_3_ NCs doped with various amounts of ethylenediammonium. (c) Energy gap (blue) between PL maxima and absorption edges and PL QY (red) as a function of ethylenediammonium loading.

In conclusion, we have developed a colloidal synthesis of monodisperse and Sn(IV)-free FASnI_3_ NCs, which allowed us to probe their intrinsic optical properties. We show that 10 nm large pure colloidal FASnI_3_ NCs exhibit an unusually large band gap, which cannot be explained solely by quantum confinement and is instead attributed to the distortion of the lattice through the split of I-sites. Such a split causes a reduction in the symmetry and bending of the Sn–I–Sn bond, reducing the overlap of Sn and I 5p orbitals. Pure FASnI_3_ NCs also exhibit a nearly featureless absorption spectrum with an absorption coefficient of only 4 ± 1.7 × 10^3^ cm^–1^ near the band gap, which might be caused by the disorder-induced smearing of the electronic structure. We also show that doping FASnI_3_ NCs with bifunctional organic cations, such as ethylenediammonium, might be an efficient way to tune the optical properties of these NCs.

## Supporting Information Available

Synthesis details; characterization techniques; WAXTS analysis; and additional notes and figures including PCE evolution, TEM images, size histograms, PL spectra, PL peak maxima, XPS spectra, TRES, photoluminescence spectra, and PIA results (PDF)
