# Supplementary material for: Intrinsic Formamidinium Tin Iodide Nanocrystals by Suppressing the Sn(IV) Impurities
Source: Nano Lett. 2023 Feb 28;23(5):1914–23. doi: 10.1021/acs.nanolett.2c04927 (PMC9999454; doi:10.1021/acs.nanolett.2c04927)
Supplement: Supplementary file 1 — nl2c04927_si_001.pdf [file nl2c04927_si_001.pdf]

Supplementary information for:

Intrinsic formamidinium tin iodide nanocrystals by suppressing the Sn(IV) impurities

Dmitry N. Dirin,[a,b*] Anna Vivani,[c] Marios Zacharias,[d] Taras V. Sekh,[a,b] Ihor Cherniukh,[a,b] Sergii Yakunin,[a,b] Federica Bertolotti,[c] Marcel Aebli,[a,b] Richard D. Schaller, [e,f] Alexander Wieczorek,[b] Sebastian Siol,[b] Claudia Cancellieri,[b] Lars P.H. Jeurgens,[b] Norberto Masciocchi,[c] Antonietta Guagliardi,[g] Laurent Pedesseau,[d] Jacky Even,[d] Maksym V. Kovalenko,[a,b] Maryna I. Bodnarchuk[a,b]

[a] Institute of Inorganic Chemistry, Department of Chemistry and Applied Biosciences, ETH Zürich, CH-8093 Zürich, Switzerland

[b] Empa-Swiss Federal Laboratories for Materials Science and Technology, CH-8600 Dübendorf, Switzerland

[c] Dipartimento di Scienza e Alta Tecnologia & To.Sca.Lab, Università dell'Insubria, 22100 Como, Italy

[d] Univ Rennes, INSA Rennes, CNRS, Institut FOTON, Rennes F-35000, France

[e] Center for Nanoscale Materials, Argonne National Laboratory, Lemont, Illinois 60439, USA

[f] Department of Chemistry, Northwestern University, Evanston, Illinois 60208, USA

[g] Istituto di Cristallografia & To.Sca.Lab, Consiglio Nazionale delle Ricerche, 22100 Como, Italy

* Dmitry N. Dirin, ddirin@ethz.ch

Table of contents

1. Chemicals 2
2. Synthesis of precursors 2
3. Synthesis of $FASnI_3$ NCs 2
4. Characterization 2
5. WAXTS analysis 4
6. XPS and HAXPES experiment and analysis 7
7. Computational details 8
8. Supplementary note 1: Synthesis of Sn(IV)-free $FASnI_3$ NCs 9
9. Supplementary note 2: treatment of the as-synthesized $FASnI_3$ NCs with various reducing agents 15
10. Supplementary note 3: absorption coefficient of intrinsic $FASnI_3$ NCs 18
11. Supplementary note 4: Transient absorption 19
12. Supplementary note 5: doping $FASnI_3$ NCs with small A-site or bifunctional cations 20
13. Supplementary Figures 21
14. Supplementary references: 31

1. Chemicals

Formamidine acetate (FAAc, Aldrich, 99%), ethylenediamine (Fluka), hydroiodic acid (57 % with stabilizer, ABCR), trioctylphosphine (TOP, 97%, STREM), diethyl ether (99.5%, Aldrich), ethanol extra dry (Acros), acetonitrile anhydrous (Acros), $LiAlH_4$ (Aldrich), diisobutylaluminium hydride (DiBAl, 1M in toluene, Aldrich), borane-tetrahydrofuran complex (1M solution in THF), $NaBH_4$ (Aldrich), hydrazine (Gerling Holz), tin fluoride (99% Acros), triethyl phosphite (TEP, Aldrich), 2,6-Ditertbutyl-p-cresol (butylated hydroxytoluene, BHT, Fluka), KI (99%, VWR) have been used as received.

SnI_2 (99%, STREM) has been purified by sublimation in ultrahigh vacuum (10^{-7} mbar, 380 °C) following by washing in anhydrous acetone for 30 minutes and drying under vacuum (5 mbar) for 1 hour.

Oleylamine (95%, STREM) has been purified by fractional sublimation.

DowTherm A (Aldrich) and oleic acid (OA, Aldrich, 90%) have been dried at 10^{-2} mbar for 1 hour, 80 °C and 120 °C correspondingly, and stored inside N_2-filled glovebox.

2,3,5,6-tetramethyl-1,4-bis(trimethylsilyl)-1,4-diaza-2,5-cyclohexadiene (Mashima's reagent) has been kindly provideded by Prof. Christophe Coperet (ETHZ).

2. Synthesis of precursors

2.1 Formamidinium oleate (FAol) was synthesized as follows. Formamidine acetate, 0.26 g (2.5 mmol), 8 mL DowTherm A and 2 mL (6.3 mmol) oleic acid were added to a 25 mL round bottom flask. The mixture was degassed for 10 minutes at room temperature, then heated under nitrogen to 130 °C yielding a clear solution of 0.25 M, and then dried for 30 min at 50 ºC under vacuum (~10^{-1} mbar).

2.2 Ethylenediammonium diiodide (EDA) was synthesized following,[1] with slight modification at the washing step. 0.668 ml ethylenediamine and 3.3 ml hydroiodic acid were reacted in a centrifuge tube in an ice bath at a molar ratio (10:25 mmol). Excess HI was added to ensure complete reaction. A yellowish powder precipitated in the tube. This powder was separated by centrifugation and washed twice with 10 ml diethyl ether, and then twice with redispersion in 20 ml ethanol followed by precipitation with 20 ml diethyl ether. The final supernatant is nearly colorless, and the powder is off-white. The obtained powder was dried in a vacuum oven for 3 hours at 50 °C. The final powder was stored inside the glovebox.

3. Synthesis of $FASnI_3$ NCs

All glassware was dried at 130 C for at least an hour and put directly inside the glovebox. Flask was loaded with 0.15 g (0.4 mmol) SnI_2 (purified by sublimation and washing), 0.4 mL TOP, 9.6 mL dried Dowtherm A, 80 uL distilled OLAm and connected to a Schlenk line without exposing materials to air. All parts outside the flask were purged three times with N_2. Then the flask was heated to 80 C under N_2 and 0.8 ml of dried FAol solution was injected. The reaction mixture was quenched by an ice bath immediately after injection and brought to the glovebox for washing without exposing NCs to air. The crude solution was centrifuged at 12.4 krpm for 10 minutes. The precipitate was redispersed in 1 ml toluene and centrifuged at 3 krpm for 2 minutes keeping only the supernatant. Obtained NCs were precipitated by anhydrous acetonitrile and redissolved in toluene.

4. Characterization

4.1 UV/vis/IR absorption spectra were collected using a Jasco V670 spectrometer.

4.2 Room-temperature steady-state photoluminescence (PL) spectra were acquired on the Fluoromax-4 Horiba spectrofluorometer equipped with a photomultiplier tube (PMT) as a detector and 150 W Ozone-free xenon arc lamp as a light source. All spectra were corrected for the detector sensitivity.

4.3 Time-resolved emission spectra (TRES) were acquired with a PicoQuant FluoTime 300 spectrometer that was equipped with a TimeHarp 260 PICO counting unit. An excitation wavelength of 355 nm was utilized (from a frequency-tripled, picosecond Nd:YAG laser by PicoQuant).

4.4 Time-resolved PL spectra were taken from TRES data for wavelengths corresponding to PL maximum.

4.5 Absolute photoluminescence quantum yield (PL QY) of NCs was acquired from colloidal solutions using the Quantaurus-QY Absolute PL quantum yield spectrometer (C11347-11; Hamamatsu) equipped with an integrating sphere. Excitation source emission wavelength was set to 450 nm.

4.6 Scanning Transmission Electron Microscopy (STEM)

DF STEM images were also recorded using JEOL JEM2200FS microscope equipped with a Schottky field emission gun operated at 200 kV.

4.7 Powder X-ray diffraction (XRD) was performed using a STOE STADI P diffractometer, operating in transmission mode. A germanium monochromator, Cu Kα1 irradiation and a silicon strip detector (Dectris Mythen) were used.

4.8 Powder XRD patterns of nanocrystals was performed with Stoe & Cie IPDS II diffractometer with an image plate detector, Cu-Kα1 radiation (graphite monochromator, λ = 1.54186 Å) and with in-house modifications (beamstop, sample holder).

4.9 Wide-angle X-ray total scattering (WAXTS) data were collected at the X04SA-MS beamline of the Swiss Light Source (Paul Scherrer Institut, Villigen, CH)[2] in form of colloidal solutions in toluene inside a spinning capillary with diameter of 0.5 mm. A borosilicate glass capillary (Hilgenberg GmbH G14) was filled with the sample and sealed under inert atmosphere, in order to avoid oxidation of Sn(II). The scattering signal of pure toluene, used as the $FASnI_3$ NCs solvent, was collected separately. The beam wavelenght λ=0.563770 Å (22 keV), was precisely determined using the NIST SRM640d silicon standard. WAXTS data were collected using a 1D MYHTEN II detector working in single photon counting mode with angular resolution of 0.0036°[3] in the 0.07-19.70 $Å^{-1}$ Q-range. Subtraction of the scattering signals of the empty capillary and air and angle-dependent absorption corrections[4] were applied to the raw data.

4.10 ^{119}Sn solid state nuclear magnetic resonance (ssNMR) was performed on a 11.7 T Bruker Avance IIIHD spectrometer (Bruker Biospin, Fällanden, Switzerland). The instrument was equipped with a 2.5 mm double-resonance MAS probe operating at 20 kHz MAS. The spectral frequency was set to 186.5 MHz. The sample temperature was set to 298 K. Spectra were acqruired using a Hahn-echo sequence with a 90° excitation pulse (3.25 μs), an echo delay of 45.125 μs and a recycle delay of 1 s. The spectra were referenced to $SnMe_4$.

4.11 Transient absorption (TA) spectroscopy with circularly-polarized pump-probe photons was performed with 325 nm pulse generated through an optical parametric amplifier using a Ti:sapphire femtosecond laser using a Helios Transient Absorption Spectrometer (Ultrafast Systems). Analysis of TA data has been made analogously to refs.80-81 in the main text.

4.12 Inductively coupled plasma mass spectrometry (ICP-MS) analysis was performed at Bachema AG. The NCs have been digested separately for Sn analysis (acidic digestion with aqua regia) and for I analysis (basic digestion with tetramethylammonium hydroxide, TMAH). Acidic digestion is not suitable for the determination of iodine as it causes iodine losses as volatile hydrogen iodide even at room temperature.[5] Basic digestion with TMAH in sealed digestion vessels at low temperature has been shown helpful to prevent iodine losses during the digestion for the ICP-MS analysis.[6] Therefore, for the determination of iodine NCs have been digested with 0.5 % aqueous solution of TMAH, according to DIN EN 15111 protocol for the determination of trace amounts of iodine by ICP-MS.

Analysis has been performed twice on a different samples. $FASnI_3$ NCs for ICP-MS analysis have been synthesized as described above but on a four times larger scale (starting from 1.6 mmol SnI_2, all other components scaled up proportionally, without changing concentrations). The final pellet, obtained after washing with acetonitrile, was redissolved in 4 ml anhydrous toluene. 0.3 ml fractions of this solution were used for Sn and I analysis. Toluene from both fractions has been evaporated under 5 mbar vacuum and ~50 °C heating. The first obtained pellet has been digested with 10 ml aqua regia (concentrated HNO_3:HCl 1:3) at 60 °C. The second obtained pellet has been digested with 12 ml TMAH solution prepared in advance by diluting 2 ml of TMAH (25 %w/w) with 10 ml of deionized water.

sample	Sn, mg/L	I, mg/L	I:Sn
#1	45.7	518	10.6
#2	74.3	1220	15.4

5. WAXTS analysis

WAXTS data were analysed by the Rietveld method (using Topas software),[7] and a modeling approach based on the computation of the DSE (using the Debussy Suite of programs).[8] The solvent scattering trace was subtracted from $FASnI_3$ data for Rietveld refinement, while it was included as an additional model component for DSE modeling.

The cubic $Pm\bar{3}m$ structure for $FAPbI_3$ reported in ref.[9] (refined from neutron diffraction data) was used as a starting structural model for $FASnI_3$, by substituting Pb with Sn. Iodide ions displacement of 0.36 Å was determined by conven-tional Rietveld refinement, relaxing iodide fractional coordinates in the direction orthogonal to the pristine linear Sn-I-Sn angle, and it was used as a fixed parameter in the DSE-based refinements. Formamidinium is included in the model as a highly disordered ion represented by 6 equivalent C atoms, each with fractional s.o.f.=0.17, and 24 equivalent N atoms, each with fractional s.o.f.=0.083. B(C) and B(N) isotropic Debye-Waller factors are fixed at value of 2 $Å^2$. All the model parameters are reported in Table WAXTS1.

According to the DSE method, a monovariate population of cubic shaped nanocrystals was built, consisting of atomistic model of the $FASnI_3$ defective crystal structure resulting from the Rietveld analysis. The DSE signal was calculated as the average differential cross section of randomly oriented nanocrystals, from the distribution of the interatomic distances.[10] Model parameters were refined against the experimental WAXTS data in the reciprocal space. The average cube edge of 7.82 nm and its size distribution of 21.66 %, were derived using a lognormal function, in good agreement with the statistical analysis of the NCs surface area carried out on TEM images of the sample (see Figure SN1F7). A bivariate population of nanocrystals (accounting for a possible anisotropic NCs morphology) does not provide any improvement of the model. Isotropic Debye Waller factors were refined independently for iodine and tin atomic species, and the large B(I) factors of 6.2 $Å^2$ and 6.6 $Å^2$ obtained from Rietveld analysis and DSE modeling for the archetypal cubic structure, are lowered to 2.8 $Å^2$ and 2.9 $Å^2$, respectively, using a cubic structure with disordered iodine ions. Concomitantly, the drop of Goodness of fit (GoF=$\sqrt{\chi^2}$) values from 0.41 to 0.38 in Rietveld analysis, and, analogously, from 1.19 to 1.08 in DSE modeling, is recorded when the iodide local displacement is introduced in the cubic model. The two fits are visually compared in Figure WAXTS1.

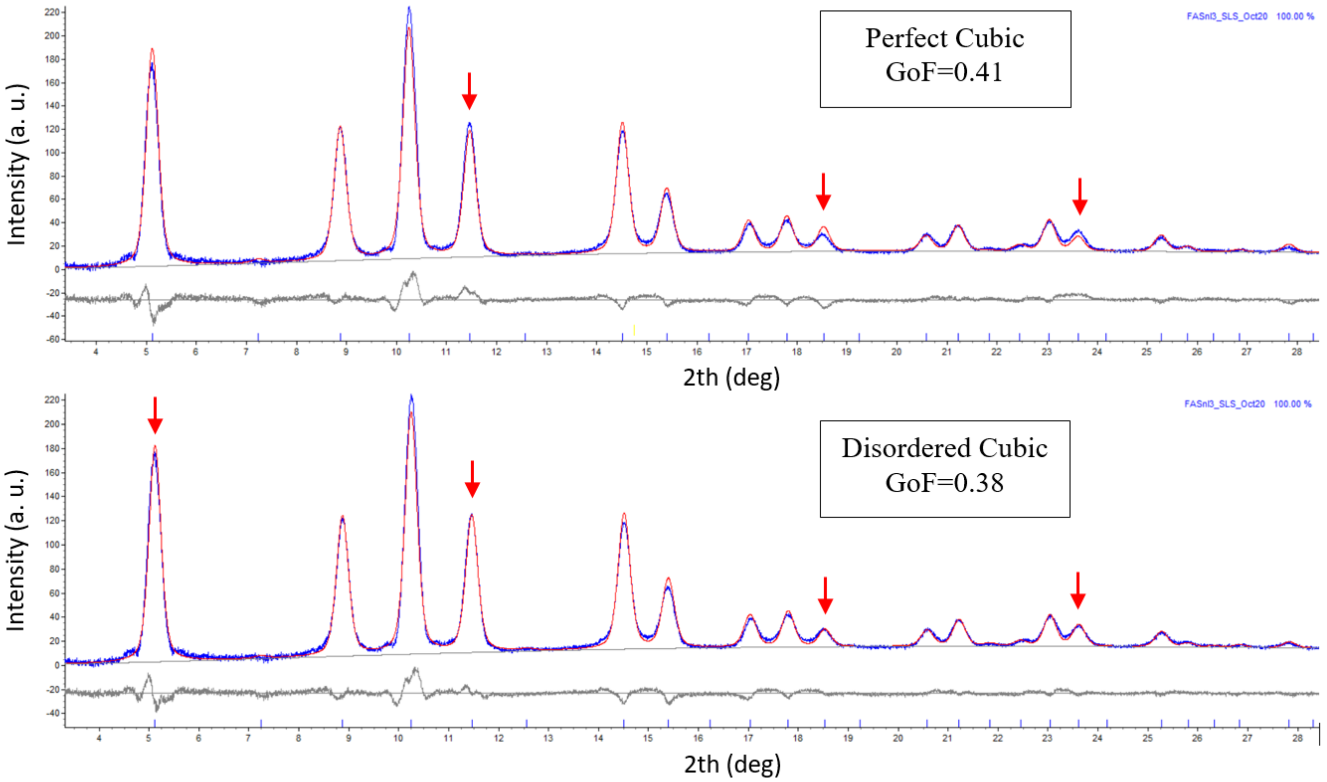

Figure WAXTS1. Rietveld refinements of the archetypal and disordered cubic models with residual differences and GoF values. The lower GoF value for the disordered model is a consequence of the better agreement between the calculated and experimental signals, on selected intensities marked by red arrows, with respect to the archetypal cubic structure.

Table WAXTS1. Refined parameters of $FASnI_3$ nanocrystals structural model with disordered iodide positions.

Formula	$FASnI_3$
Crystal system	Cubic
Space group	*Pm-3m* (No. 221)
a (Å)	6.3119(1)
V (Å^3)	251.47(2)
Sn, xyz, s.o.f., B_{iso} (Å^2)	0.0, 0.0, 0.0, 1, 4.27(5)
I, xyz, s.o.f., B_{iso} (Å^2)	0.5, 0.0, 0.0565(4), 0.25, 2.81(6)
C, xyz, s.o.f., B_{iso} (Å^2)	0.5, 0.5726, 0.5, 0.17, 2.00
N, xyz, s.o.f., B_{iso} (Å^2)	0.682, 0.5781, 0.5, 0.0833, 2.00

Table WAXTS2. Rietvled refinement results obtained analysing $FASnI_3$ single crystal diffraction data provided by Kahamnn et al.[11] Better agreement with diffraction data is provided using the archetypal cubic model (R1=3.92%), if compared to the two alternative models investigated, the former characterized by the displacement of only iodide positions in the plane perpendicular to the Sn-Sn direction (R1=4.14%), and the latter by the displacement of both, iodide (offset as before) and tin ions along the [111] crystallographic direction (R1=4.16%).

Parameter	Archetypal cubic	I-disordered cubic	I and Sn-disordered cubic
R1	3.92%	4.14%	4.16%
Num. of refined parameters	5	7	8
xyz(Sn)	0.5, 0.5, 0.5	0.5, 0.5, 0.5	0.51189, 0.51189, 0.51189
U(Sn)	0.03986	0.03833	0.03255
xyz(I)	0.0, 0.5, 0.5	0.0, 0.52751, 0.50	0.0, 0.52751, 0.50
U(I)	0.03372; 0.10281	0.03236; 0.08532	0.03228; 0.08528
xyz(Mn)	0.0, 0.0, 0.0	0.0, 0.0, 0.0	0.0, 0.0, 0.0
U(Mn)	0.64287	0.57915	0.57928
Sn/Sn	0	0	0.15
Sn-I	3.15	3.16	3.08

The background has been modeled in the DSE analysis by combining the solvent signal with a polynomial function, suggesting the occurrence of additional scattering materials besides the solvent. The contribution from small spherical nanoparticles (<2 nm) of different species ($FASnI_3$, FA_2SnI_6, SnI_2, SnI_4 and SnO_2), acting for eventual amorphous phases, was tested and excluded (Table WAXTS3, Figure WAXTS2). Therefore, the polynomial component used as a part of the background profile was extracted from the DSE fit and the G(r) function was calculated by means of the PDFgetX2 program[12] in the 0.5-30 Å r range using a sampling step of 0.01 Å (Q_{max}=19 Å^{-1}). Only one peak at 2.9 Å is reliable as interatomic distance and might be ascribed to solvated species (likely residual precursors). Remarkably, the peak found at 2.9 Å is not attributable to the Sn-O distance in neither SnO_2 nor the solvated Sn^{4+} ions whose PDF peaks are reported in ref.[13].

Table WAXTS3. GoF values reported for DSE best fit, without amorphous phase, and DSE fits performed with additional amorphous phases.

Amorphous Phase	GoF
**/ (Best fit)**	1.08
**$FASnI_3$**	2.92
**FA_2SnI_6**	3.13
**SnI_2**	2.86
**SnI_4**	3.29
**SnO_2**	3.41

a

b

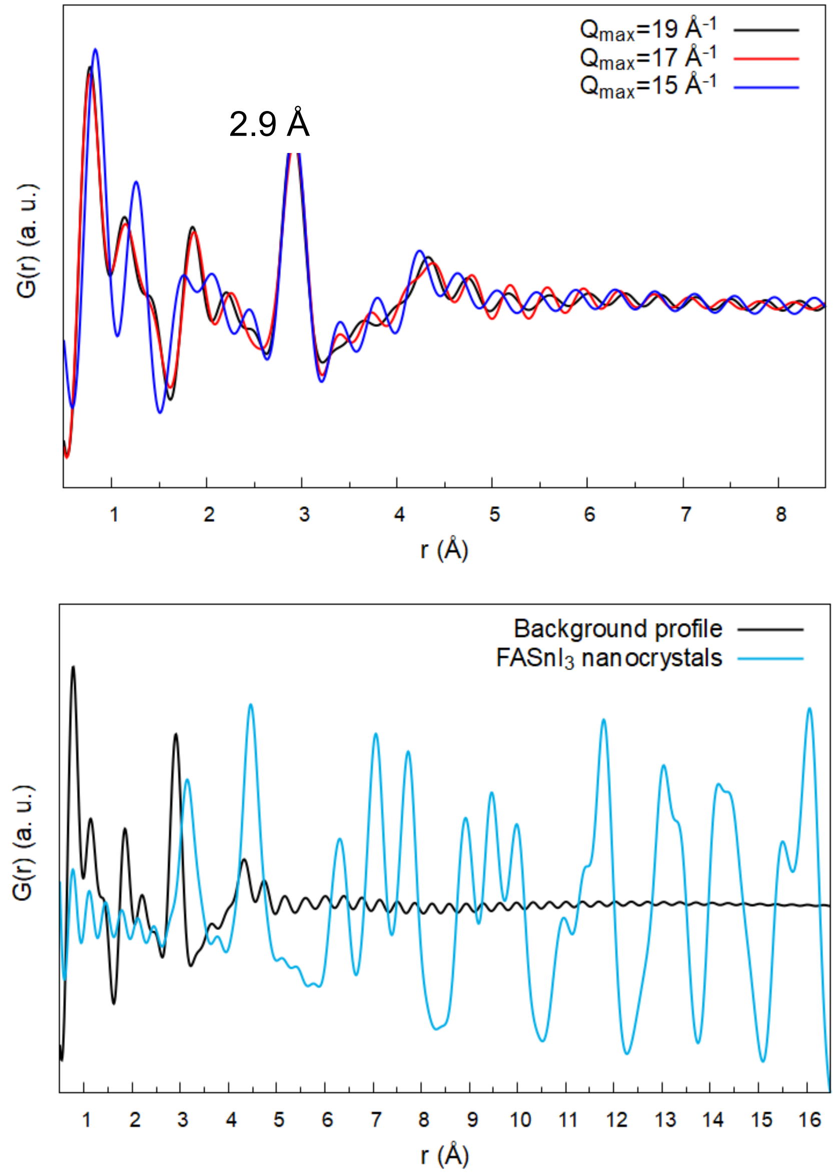

Figure WAXTS2. (a) G(r) signals obtained with values of Q_{max} of 19, 17 and 15 $Å^{-1}$, showing that the peak at 2.9 Å is not dependent on the G(r) computation. (b) G(r) signals of background profile and $FASnI_3$ nanocrystals DSE simulation are compared.

6. XPS and HAXPES experiment and analysis.

X-ray photoelectron spectroscopy (XPS) and hard X-ray photoelectron spectroscopy (HAXPES). A PHI Quantes photoelectron spectroscopy system equipped with both hard Cr-Kα and Al-Kα X-ray radiation sources was used for the combined XPS/HAXPES measurements. The take-off angle was set to 90° while a power of 100 W and voltage of 20 kV was used for the electron beam generation. A PHI Quantum system equipped with an Al-Kα monochromatic X-ray radiation source was employed for the modified Auger parameter investigation. The takeoff angle was set to 45° while a power of 12.6 W and voltage of 15 kV was used for the electron beam generation while the linearity of the energy scale was calibrated using the Au $4f_{7/2}$, Ag $3d_{5/2}$ and Cu $2p_{3/2}$ lines for the XPS instrument according to ISO #15472. To further minimize the beam damage during this specific investigation, the beam spot with a diameter of 50 μm was continuously scanned over an area of 500 × 1000 μm^2. Additionally, short-term measurements (< 3 min) of the Sn 3d core level before and after each presented measurement were conducted to rule out changes in the chemical state due to X-ray induced beam damage. All XPS and HAXPES measurements were performed at a pressure below $2\cdot10^{-6}$ Pa. Charge neutralization was achieved using a low-energy electron flood gun. The C 1s feature at 284.8 eV was used as a charge reference, resulting in a typical measurement error of ±0.2 eV, which does not affect the values of the modified Auger parameter. Peak fitting was performed after Shirley background subtraction using Voigt profiles with a GL ratio of 40. To estimate the information depth depending on the observed feature, the inelastic mean free path (IMFP) was calculated from the kinetic energy of the detected electrons based on the Tanuma, Powell, Penn formula.[14]

Conventional XPS analysis by soft Al-Kα X-rays results in a relatively small probing depth in the range of 2-6 nm and, consequently, mainly the ligand shells regions of the $FASnI_3$ NCs are probed. The N 1s photoelectron line was measured at small and large probing depths by Al-Kα and Cr-Kα X-rays, respectively. As such, the two chemical species of N in the $FASnI_3$ NCs could be unambiguously assigned to N contained in the FA cation of the perovskite structure, (N_{FA}^{+}, lower BE peak at 400.2 eV, which is more pronounced for higher probing depth) and N in oleylammonium (N_{OAm}^{+}, higher BE peak at 401.7 eV). A considerably larger average volume fraction of the NP core region is probed using Cr-Kα X-rays.

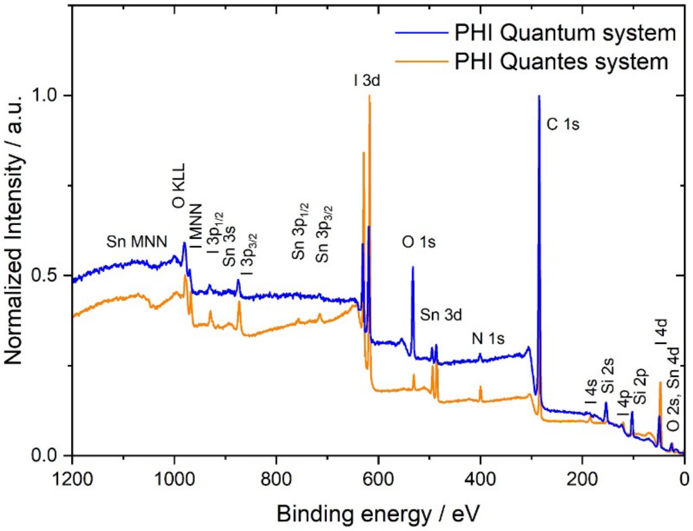

Figure XPS1. XPS survey spectra of $FASnI_3$ NC film measured with PHI Quantum system (blue line, Al-Kα X-ray source) and PHI Quantes system (orange line, Al-Kα X-ray source).

7. Computational details

All DFT calculations for $FASnI_3$ were performed using the Quantum ESPRESSO code[15-16] together with the Perdew-Burke-Ernzerhof revised for solids (PBEsol) functional[17] and optimized norm-conserving Vanderbilt pseudopotentials.[18] The plane wave energy cut-off was set to 120 Ry and the lattice constant was fixed to the experimental value.[11] For the archetypal ordered structure of FASnI[3], we employed the cubic unit-cell containing 12 atoms and placed the FA molecule either into the same orientation as calculated for the disordered structure or perpendicular to the z-axis, i.e. ignoring its random orientation. The comparison of these two orientations is shown in Figure CD1 and indicates the importance of allowing the FA molecules to explore random orientations which on average reflect the cubic symmetry. Employing a similar procedure to that described in ref.[19], we obtained the ground state structure of cubic $FASnI_3$ in a 2×2×2 supercell. The procedure consists of the following steps: (i) a DFT calculation in the ordered structure of $FASnI_3$, (ii) the computation of the phonons of the ordered structure by means of density functional perturbation theory,[20] (iii) the displacement of the atoms in a 2×2×2 supercell of the ordered structure along all phonon modes, including soft modes, populated at T =0 K,[21] and (iv) a tight geometry relaxation of the supercell until the residual force component per atom becomes less than 3×10^{-4} eV/Å. We found that the disordered structure yields a significant lowering of the total energy of 223 meV per formula unit.

The spectral function was calculated using the disordered 2×2×2 $FASnI_3$ supercell and the band structure unfolding technique[22] as implemented in the EPW code.[21, 23] We sampled the X-R-M-Γ high symmetry path using 288 equally spaced k-points. The DOS was obtained as a summation of the spectral function over all k-points. The absorption coefficient and photoluminescence spectra were evaluated fully from first principles using the real and imaginary parts of the dielectric function.[24-25]

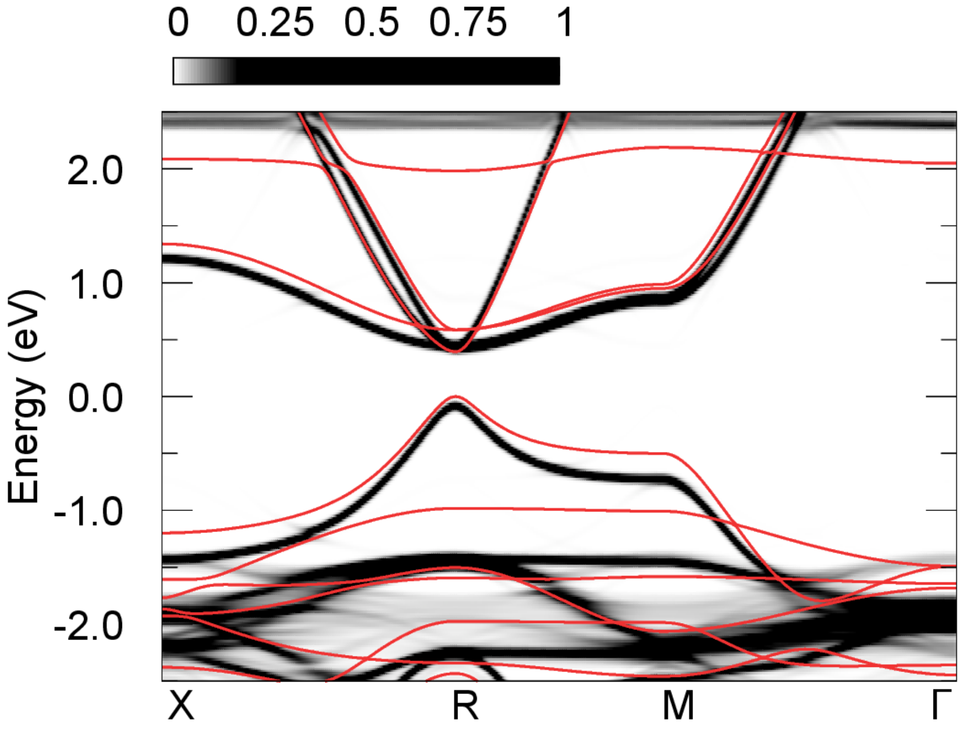

Figure CD1. Comparison of the momentum resolved electron spectral functions of the archetypal cubic structure with FA in orientations as calculated for the disordered structure (black curves) and for the case when FA is placed perpendicular to the z-axis (red line).

8. Supplementary note 1: Synthesis of Sn(IV)-free $FASnI_3$ NCs

In the footstep of our earlier reports on the synthesis of $FAPbI_3$ NCs, first edition of the $FASnI_3$ NCs involved 1-octadecene (ODE) as a solvent (Figure S2). Briefly, oleylamine (OAm), oleic acid (OA), and formamidinium oleate are sequentially injected into hot SnI_2 solution in trioctylphosphine (TOP) and ODE. However, reactions at 80 °C nearly instantly lead to the oxidation of iodide anions to polyiodide ones, apparent from their characteristic red-brown color. Although synthesis in ODE allows obtaining monodisperse NCs, we expect polyiodides to subsequently oxidize Sn(II) (*ca*. 0.4 V higher standard reduction potential in aqueous solutions). Indeed, photoluminescence (PL) spectra of these NCs do not correlate with their size, and PL maximum fluctuates in a broad range from 700 nm to 850 nm (Figure S3). We therefore opt for a synthesis in aromatic solvents (toluene, mesitylene, or Dowtherm A) that proceeds without any color change until formamidinium oleate solution is injected and NCs start to grow (Figures S4, S5).

All tested, commercially available sources of SnI_2 exhibit insufficient purity. Commercial SnI_2 forms solutions with irreproducible yellow-to-brownish tint and oftentimes is not dissolving in the reaction mixture without insoluble rests. We hypothesized that insoluble impurity might be partially oxidized SnO/SnO_2. Besides, apolar-solvent-soluble SnI_4 may contaminate the solution and remain unnoticed. Therefore, we have purified commercial SnI_2 by sublimation. The leftover after the sublimation step consists of SnO_2 (Figure SN1F1), whereas sublimed material consists of a mixture of SnI_2 and SnI_4 (Figure SN1F2). The latter was washed out by anhydrous toluene as confirmed by the removal of the corresponding peaks from X-Ray diffraction (XRD) pattern and shift of the binding energy in X-ray photoelectron spectroscopy (XPS) data (Figures SN1F3, SN1F4). We emphasize that these synthetic precautions did not significantly change the morphology of the resulting $FASnI_3$ NCs but drastically improved the reproducibility of the synthetic protocol and the resulting optical properties (Figures 1, 2 of the main text).

Synthesis performed without OAm results in large $FASnI_3$ crystals (Figure SN1F5), suggesting that oleylammonium acts as a capping ligand. Synthesis performed with tin oleate as a source of Sn was found to produce significant quantities of layered tin iodide perovskites with PL around 700 nm, which we assign to $OAm_2FASn_2I_7$ (Figures SN1F5, SN1F6).[26] Similarly, the synthesis of $CsSnX_3$ NCs performed with tin oleate is reported to produce CsX or very irregular $CsSnI_3$ NCs.[26-27] We therefore choose to avoid using the tin oleate precursor and solubilize SnI_2 with TOP. Variation of the injection temperature from 25 to 110 °C has nearly no effect on the final size and size dispersion of NCs (Figure SN1F7).

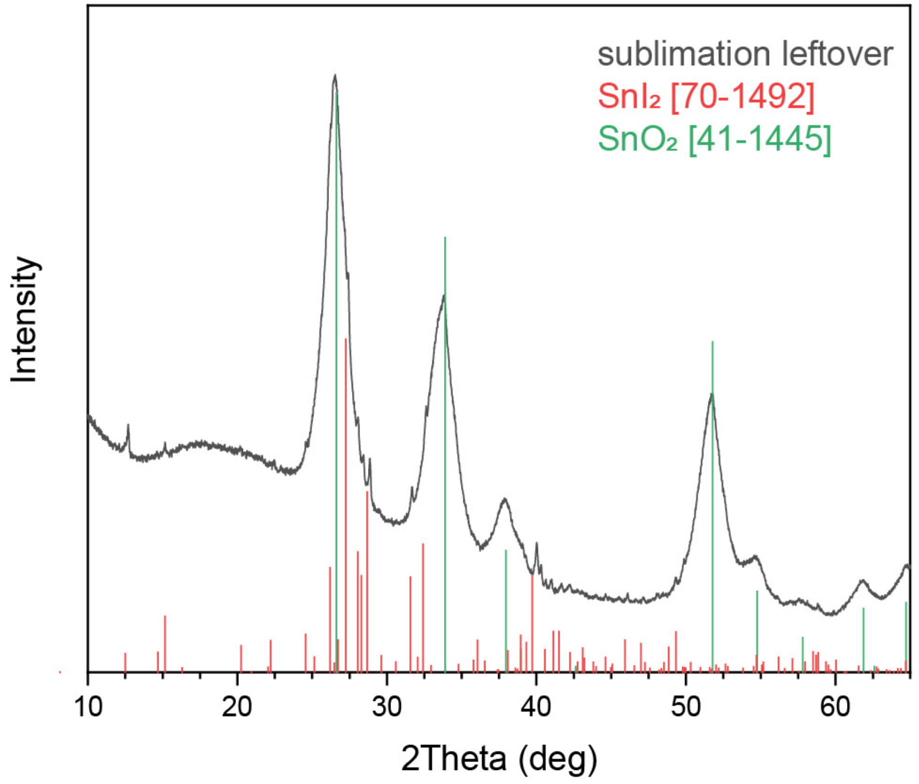

Figure SN1F1. Powder XRD pattern of the material left after sublimation of SnI_2 in ultrahigh vacuum. This pattern indicates that majority of the left material consists of nanocrystalline SnO_2.

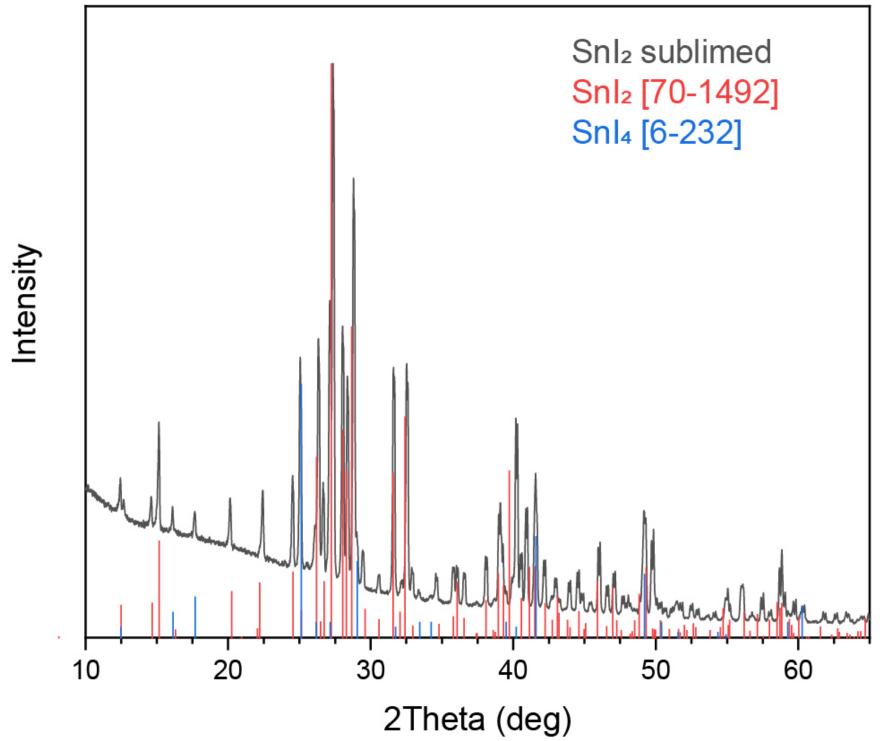

Figure SN1F2. Powder XRD pattern of the sublimed SnI_2. This pattern indicates that sublimed material consists mainly of SnI_2 but contains notable amount of SnI_4 as well.

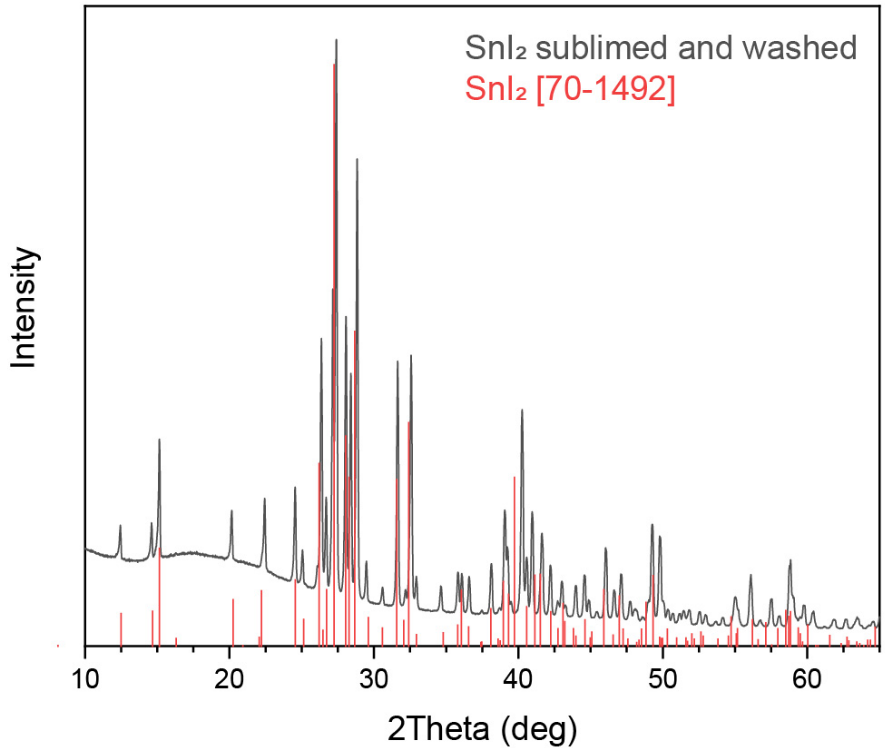

Figure SN1F3. Powder XRD pattern of the sublimed and washed commercial SnI_2 indicating crystallographically pure SnI_2.

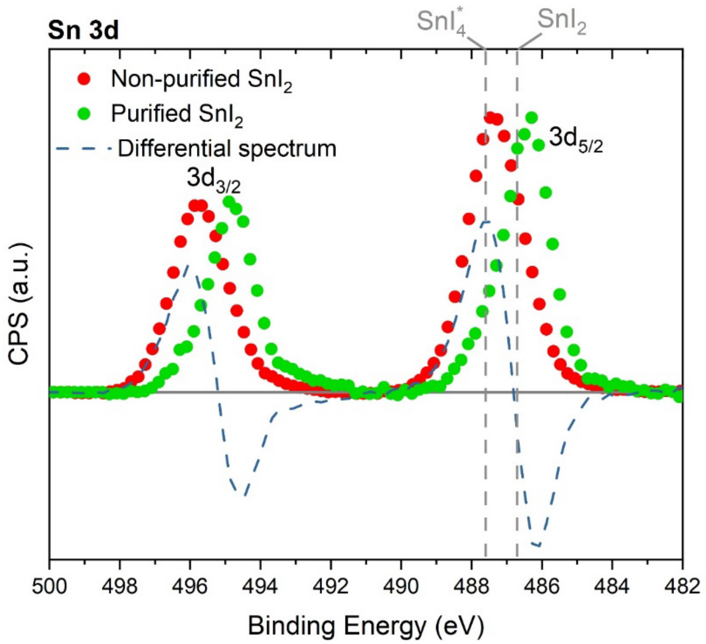

Figure SN1F4. XPS spectra of the purified and non-purified SnI_2 and reference peaks for SnI_2[28] and SnI_4. We note that there are no available references for pure SnI_4. Instead we use peak reported for the closest analogs: SnI_4 impurity in ref.[29] and reference extracted for $SnI_4(C_4H_4N_2)$ the NIST database.[30] These two references coincide at 487.6 eV.

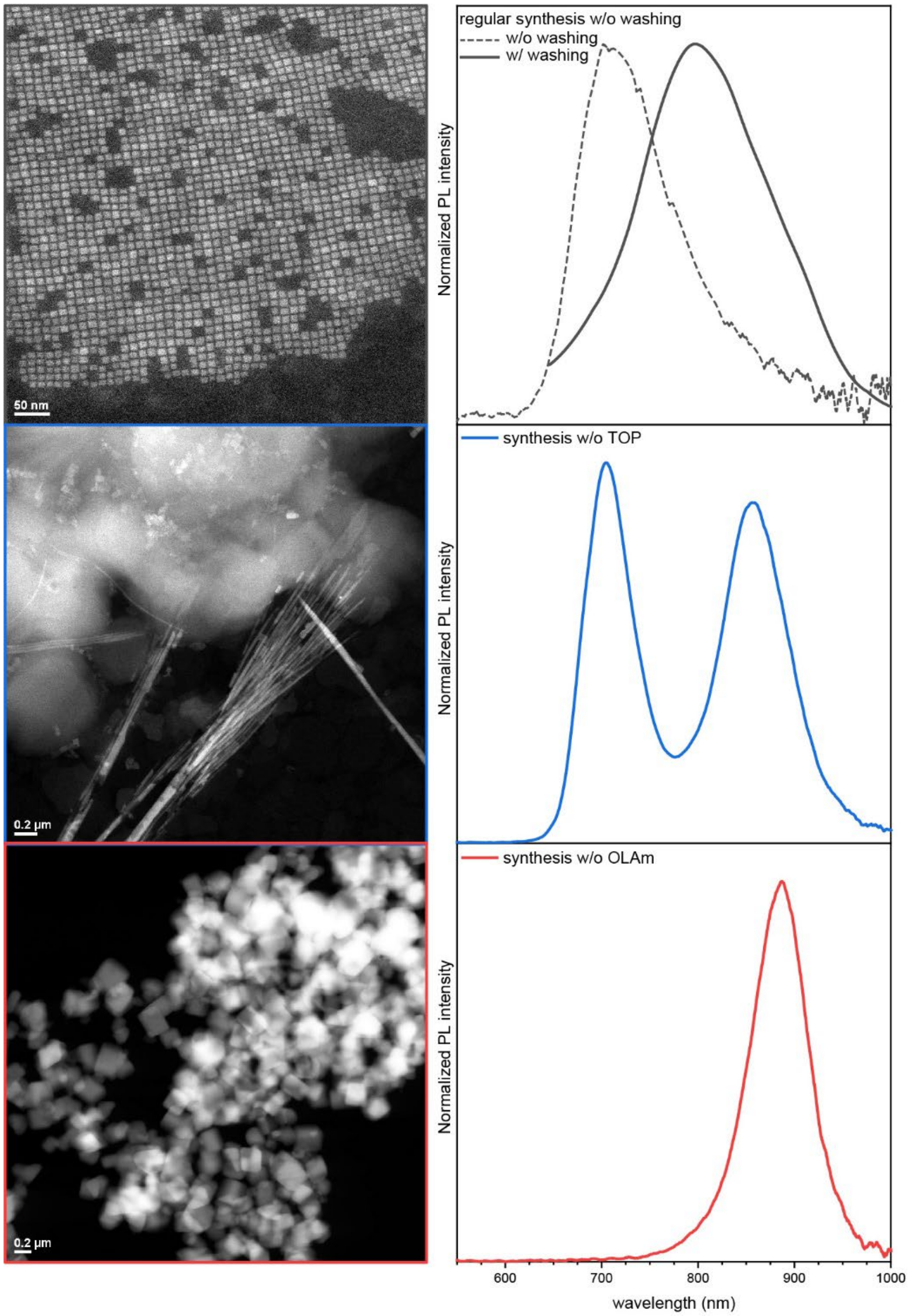

Figure SN1F5. DF STEM images of regular $FASnI_3$ NCs (upper panel, grey), NCs synthesized without TOP (middle panel, blue) and without oleylamine (bottom, red).

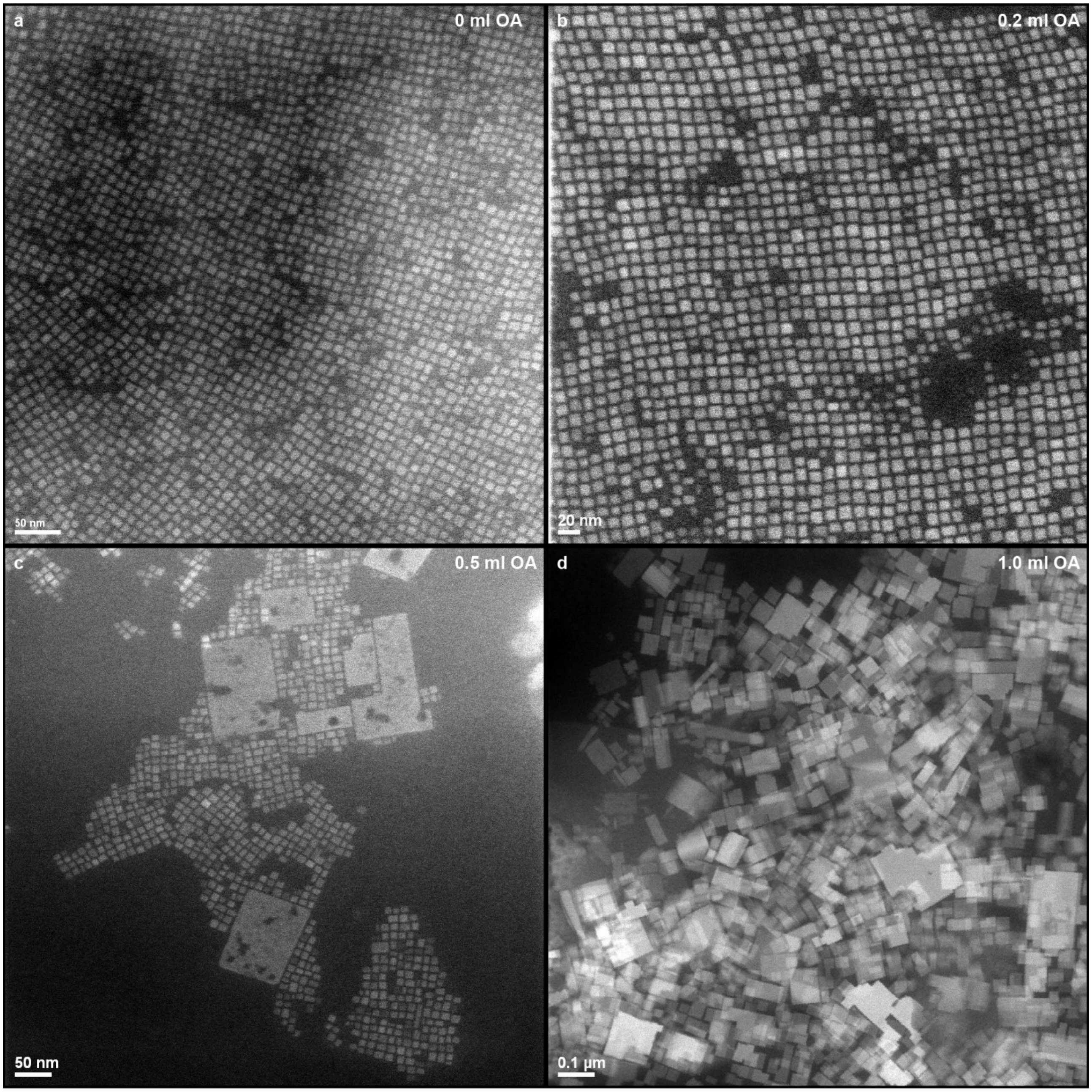

Figure SN1F6. DF STEM images of $FASnI_3$ NCs synthesized with various amounts of oleic acid (OA).

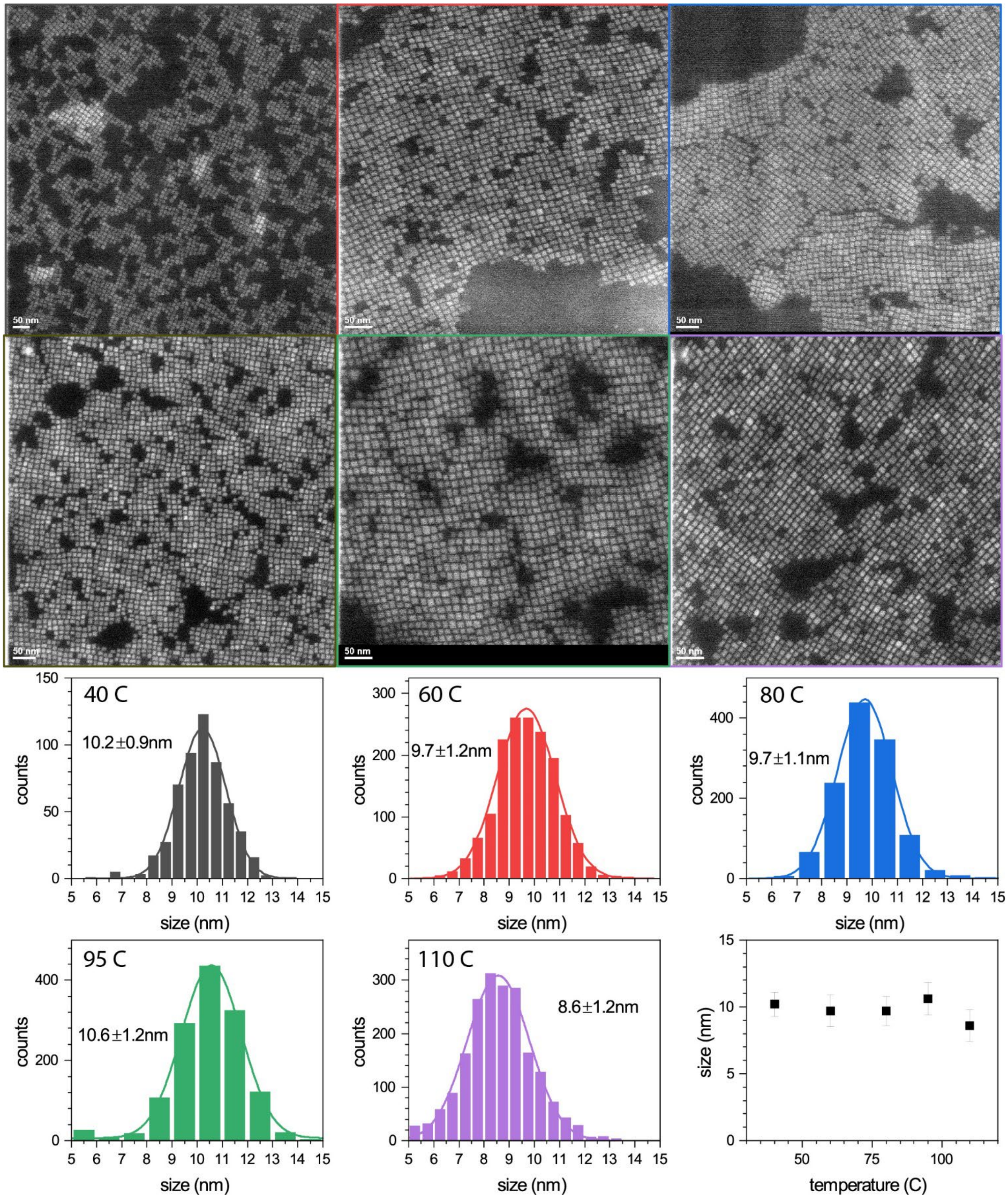

Figure SN1F7. DF STEM images of $FASnI_3$ NCs synthesized at different temperatures and corresponding histograms: 40 °C (grey) 60 °C (red), 80 °C (blue), 90 °C (brown), 95 °C (green), 110 °C (purple).

9. Supplementary note 2: treatment of the as-synthesized $FASnI_3$ NCs with various reducing agents

Even in the absence of Sn(IV) in the starting materials, this impurity may be generated by oxidation during storage of the NCs. We consider three possible redox reactions:

$$FASnI_3 \rightleftharpoons Sn^{4+} + FA^+ + 3I^- + 2e^- \qquad (1)$$

$$FASnI_3 \rightleftharpoons Sn^{2+} + FA^+ + I_3^- + 2e^- \qquad (2)$$

$$FASnI_3 + 2e^- \rightleftharpoons Sn^0 + FA^+ + 3I^- \qquad (3)$$

The standard reduction potentials of the main involved free ions are shown in Figure SN2F1a. Due to the binding of the ions in a perovskite crystal lattice, these ions are stabilized and the formal reduction potentials of these ions differ by $\frac{RT}{nF}\ln K_{SP}$, where K_{SP} is the solubility product of $FASnI_3$ in a given solvent and n=2 for all three reactions mentioned above. The actual value of K_{SP} is unknown, but we evaluate it to be as small as 10^{-10}-10^{-25} mol^5/L^5, similar to $CsPbBr_3$.[31] The blurred red bars in Figure SN2F1a indicate the range of possible formal reduction potentials of reactions (1)-(3). Even such rough and conservative evaluation shows that oxidation according to reaction (1) may be favored in the presence of photoinduced carriers and electron scavengers. Indeed, pure $FASnI_3$ NCs do not change absorption or PL properties over several months when stored air-free in ambient light but oxidize in hours when exposed to air in darkness. In contrast, even a weak visible light flux catalyzes their oxidation and allows its completion in minutes or even seconds, depending on the flux.

The air stability of the $FASnI_3$ NC films was evaluated by comparing the XPS surveys of a non-air-exposed and air-exposed (for 5 min under ambient light) film (Figure SN2F2). Interestingly, both films show only a minute O 1s surface contamination, which is unaffected by short air exposure, indicating that the films are resistant to oxidation for such short-term air exposures. However, exposure to X-rays during the XPS/HAXPES analyses does result in partial degradation of the $FASnI_3$ NC films, as evidenced by the appearance of a second component in the Sn 3d core level spectrum (Figure SN2F3), as well as a change in the shape of the background of inelastically scattered photoelectrons (Figures SN2F4, SN2F5). Notably, combined lab-based XPS/ HAXPES analysis indicates that this degradation is most pronounced in the surface region (Figure SN2F3).

To probe the presence of Sn(IV) impurity, we treat $FASnI_3$ NCs with reducing agents of very different strength and concentrations that can be added either to the product or during the synthesis (Table SN2). We also check the effect of KI which could fill potentially present I vacancies and thus stabilize $FASnI_3$ against oxidation. Furthermore, to ensure the selective reduction of Sn(IV) but not Sn(II), we use the pair of SnF_2 with Mashima's reagent applied recently in $FASnI_3$ photovoltaics.[32] This reagent is highly selective to SnF_2 and reduces it in situ to metallic Sn, which in its turn, can reduce potential impurities of Sn(IV). In all these cases, the treatment resulted in either complete reduction of Sn(II) to metallic Sn(0) (in case of strong reducing agents at high concentrations) or did not affect the optical properties of $FASnI_3$ NCs (mild reducing agents, strong reducing agent at low concentrations, and all other tested cases), as summarized in Figure SN2F1b. Together with the insignificant level of Sn(IV) detected in XPS and ^{119}Sn NMR, this indicates that the amount of Sn(IV) impurities in the synthesized $FASnI_3$ NCs is extremely low and does not affect the optical properties of the obtained NCs.

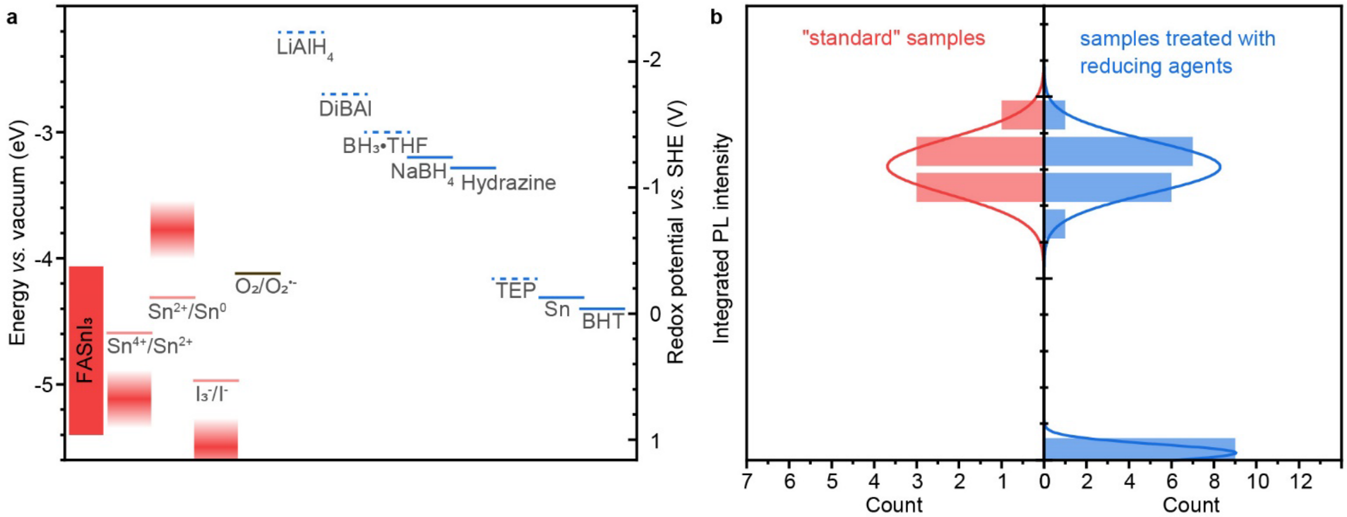

Figure SN2F1. (a) The bandgap of $FASnI_3$ in comparison with the standard reduction potentials of the relevant species and reducing agents used in this work; the areas with gradient filling indicate possible formal redox potentials of Sn(IV), Sn(0), and I^{3-} according to reactions (1)-(3) in the main text; for some species the precise reduction potentials are not available in the literature, and therefore their estimated values are indicated as dashed blue lines. (b) An integrated PL intensity for a series of regular $FASnI_3$ NCs (left panel, red) and samples synthesized or treated with various reducing agents (right panel, blue).

Table SN2. The summary for the reducing and other treatment agents used in this work with their properties and the obtained result.

#	treating agent	properties	result
1	$LiAlH_4$	a very strong nucleophilic reducing agent	fast (<1 min) and complete reduction to metallic Sn at concentrations above 0.01 eq.<br>no effect on PL and slow (several hours) precipitation of NCs at lower concentrations
2	DiBAl*	a strong electrophilic reducing agent	fast (<1 min) and complete reduction to metallic Sn at concentrations above 0.01 eq.<br>no effect on PL and slow (several hours) growth and precipitation of NCs at lower concentrations
3	BH_3•THF	intermediate-strong reducing agent	progressive growth and precipitation of NCs without any notable change in PL intensity at concentrations of 10^{-3}-10^{-1} eq.
4	$NaBH_4$	intermediate power reducing agents	fast (<1 min) and complete reduction to metallic Sn at concentrations above 0.01 eq.<br>no effect on PL and slow (several hours) precipitation of NCs at lower concentrations
5	Hydrazine		
6	TEP	mild antioxidants, can be added either to the product or during the synthesis	no effect
7	BHT		
8	SnF_2 + Mashima's reagent	Mashima's reagent selectively reduces SnF_2, creating in situ Sn(0) which can react with potential Sn(IV) impurities, if present	no effect below 0.01 eq and quick precipitation at concentrations in range 0.01-1 eq.
9	KI	may fill V_I, if present	no effect
10	SnF_2	mild reducer; can be added either to the product or during the synthesis	no effect on PL of the final NCs;<br>when added during the synthesis it reduces polyiodides if present in the solution

* DiBAl – diisobutylaluminium hydride; TEP – triethyl phosphite; BHT – butylated hydroxytoluene; Mashima's reagent – 2,3,5,6-tetramethyl-1,4-bis(trimethylsilyl)-1,4-diaza-2,5-cyclohexadiene

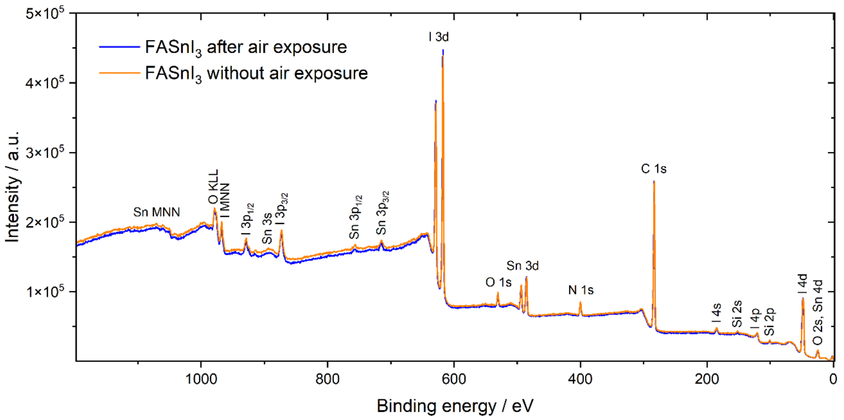

Figure SN2F2. XPS survey spectra of $FASnI_3$ NC films with (blue line) and without (orange line) air exposure.

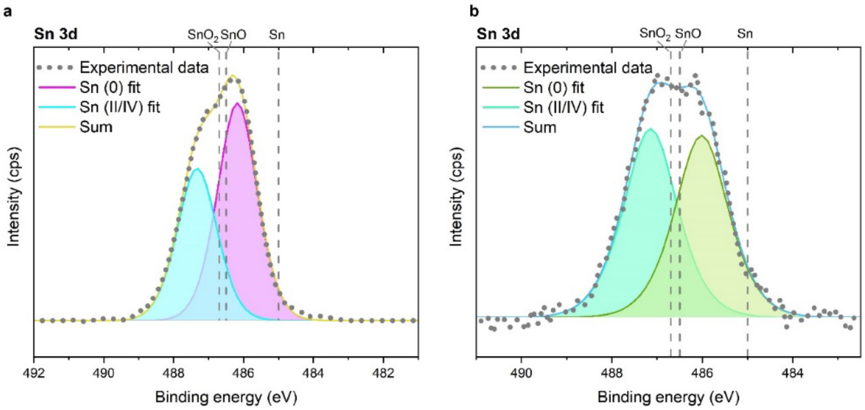

Figure SN2F3. a) XPS Sn 3d spectrum for $FASnI_3$ NC films measured with Al-Kα X-Ray source. In contrast to other herein presented measurements of the Sn 3d region, higher X-Ray powers were used. b) Respective HAXPES spectrum measured with Cr-Kα X-ray source. Reference peaks for Sn, SnO and SnO_2 were extracted from the NIST database.[30] The films were partly damaged upon the X-ray exposure in the HAXPES experiment resulting in the change of the oxidation state of Sn and the appearance of the feature corresponding to Sn(0). This peak appears to be of higher intensity for the spectrum made with Al-Kα X-ray source (58.9 % of total Sn 3d feature intensity) than for the one made with Cr-Kα X-ray source (49.1 % of total Sn 3d feature intensity). This finding is in agreement with our notion that NCs in the uppermost layers are the ones that are most prone to degradation upon X-ray exposure (visible with Al-Kα X-ray source) and deeper lying NCs are less affected.

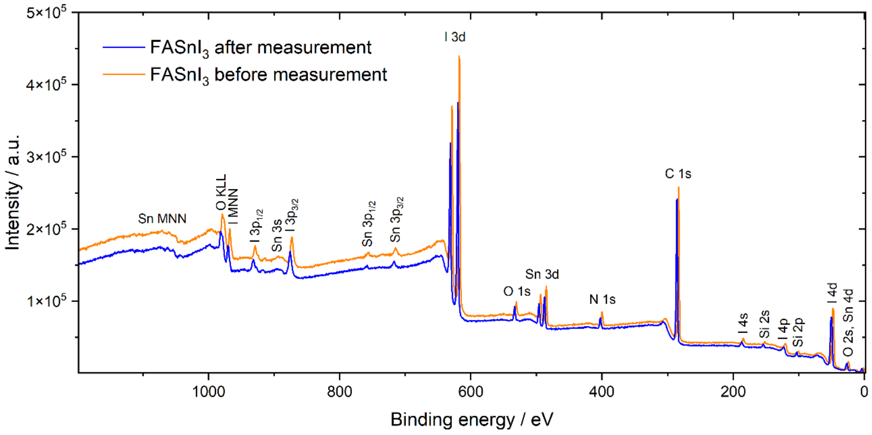

Figure SN2F4. XPS survey spectra of $FASnI_3$ NC films before (orange line) and after (blue line) the combined XPS/HAXPES measurement.

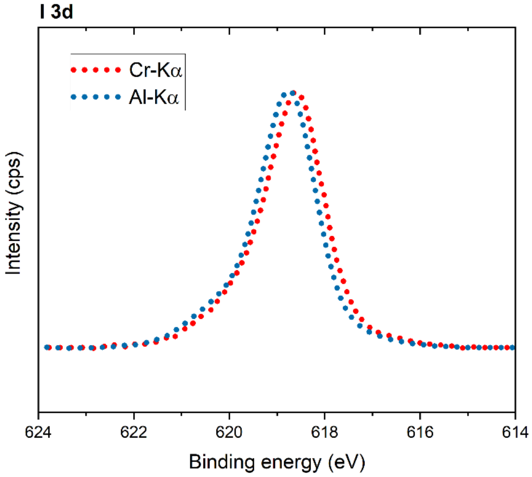

Figure SN2F5. XPS I 3d spectra for $FASnI_3$ NC films made with a) Al-Kα (blue dots) and b) Cr-Kα (red dots) X-ray sources. Unlike in the case of Sn, the oxidation state of I seems to be barely affected by the NC degradation due to the high degree of resemblance between I 3d spectra at different probing depths.

10. Supplementary note 3: absorption coefficient of intrinsic $FASnI_3$ NCs

Absorption spectra of two concentrated solutions of pure $FASnI_3$ NCs in hexane are shown below. Concentration of $FASnI_3$ (without ligands) according to ICP-MS is 0.21 and 0.34 mg/ml for black and red spectra respectively. In this case the molar concentration of 9.7 nm large NCs is 0.11-0.17 μM and molar extinction coefficient at 750 nm (100 meV above the bandgap of NCs, optical density 0.132-0.118 cm^{-1}) is $\varepsilon \sim 9.6\pm4.0\cdot10^5$ $M^{-1}cm^{-1}$. This value can be recalculated to intrinsic absorption coefficient as following:[33]

$$\mu_i = \frac{\varepsilon \ln 10}{N_A d^3}$$

The obtained intrinsic absorption coefficient is ~$4.0\pm1.7\cdot10^3$ cm^{-1}, which is about four times smaller than that one of bulk $FASnI_3$.[34]

sample	$FASnI_3$ w/o ligands, mg/ml	[NCs], M	ε, $M^{-1}cm^{-1}$	μ, cm^{-1}
#1	0.2096	1.06E-7	$12.454\cdot10^5$	$5.22\cdot10^3$
#2	0.3408	1.72E-7	$6.828\cdot10^5$	$2.86\cdot10^3$
average			$9.6\pm4.0\cdot10^5$	$4.0\pm1.7\cdot10^3$

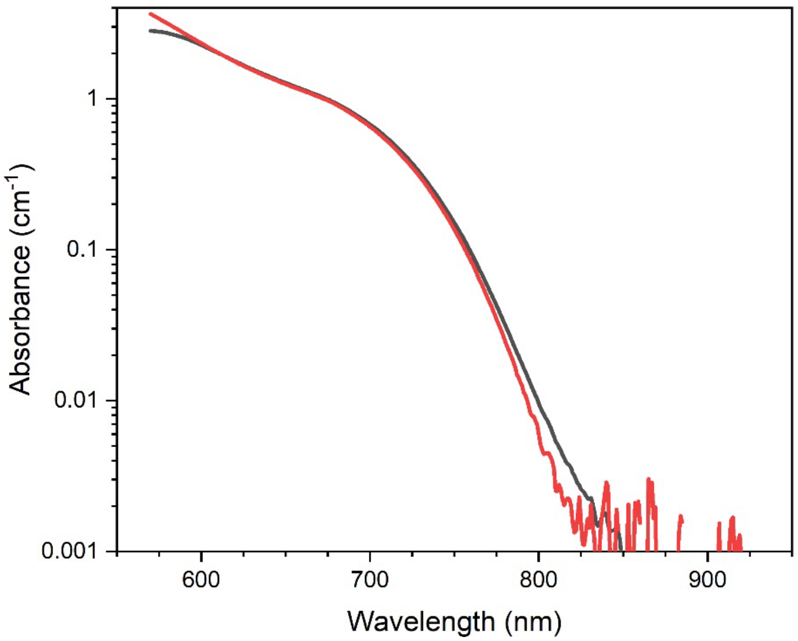

Figure SN3F1. Absorption spectra of $FASnI_3$ NCs solution in hexane used for the measurement of extinction coefficient. Optical path is 1 cm.

11. Supplementary note 4: Transient absorption

Figure SN4F1a shows spectrally-resolved transient absorption (TA) data of $FASnI_3$ NCs pumped at 400 nm, well above the bandgap. This spectral map reveals two pronounced bleaches around 600 and 525 nm. Both bleaches are accompanied by depopulation of the higher-lying states and appear at about 5 ps, on par with the cooling time of hot carriers in bulk $FASnI_3$.[35] Note that the lowest-lying observed bleach in Figure SN4F1a (600 nm) is still 0.5 eV above the band edge of absorption (Figure 2a). Following the recent data on metal halide perovskite NCs and films, we ascribe this bleach to the VB1-CB2 transition where CB1 and CB2 are SOC-splitted conduction band states (Figure SN4F1b).[36-37] The detailed description of the electronic structure of $FASnI_3$ NCs can be found in ref. [37] The lowest-lying VB1-CB1 transition (corresponding to B1 bleach) and the second VB1-CB2 transition (B2 bleach) in bulk $FASnI_3$ are reported to be at 800-900 nm and 660 nm, respectively.[37] The energy gap between these two bleaches, as well as B1 and B2 bleaches for $CsSnBr_3$ is about 0.42 eV apart and coincides with the SOC-induced CB splitting (Δ_{SO}) calculated for tin halide perovskites.[38-39] Altogether, this indicates that the bleach observed at 600-620 nm corresponds to the VB1-CB2 transition. The bandgap bleach (B1) could not be resolved due to a weak oscillator strength: optical density 100 meV above the band edge was only about 0.1 cm^{-1}, even for solutions with the highest feasible concentration of NCs.

Figure SN4F1c shows the comparison of TA spectra with circularly co- ($\sigma^+\sigma^+$, $OD_\parallel$) and cross-polarized ($\sigma^+\sigma^-$, $OD_\perp$) pump/probe photons.[40-41] Net spin relaxation can be calculated as the difference between co- and cross-polarized TA signals and is insensitive to the specific probe wavelength at B2 (Figure SN4F1d).[37, 40] The net spin relaxation kinetics is fitted using a single-exponential decay and yields a lifetime of 2 ps, on par with the one of LHP NCs (~1-3 ps), which is much shorter than in case of $CsSnBr_3$ NCs.[37]

In addition to the expected bleaches, Figure SN4F1 reveals a pronounced photo-induced absorption (PIA) band below the B2 bleach (Figure SN4F1a, SN4F1c). Depending on the experimental conditions, this PIA band occurs on different timescales and at slightly different energies. In the case of resonance pumping (Figure SN4F1c, S9) the PIA band is polarization-sensitive, appears at ~0.2 ps, has a maximum at 703 nm, correlates with depopulation of B2, and can be explained as a biexciton shift.[37, 40] In the case of pumping at 400 nm (Figure SN4F1a, S9), in contrast, PIA appears before the thermalization of carriers to B2 state, has a maximum around 670 nm, and is also significantly more intense. This type of PIA is nearly absent with resonance pumping. We suggest that this PIA can arise due to the additional photo-induced states created upon lattice distortion induced by hot carriers. Fast PIA to these states becomes possible until the hot carrier is thermalized and lattice distortion is released. Note, that such strong PIA was not observed for $CsPbX_3$.[40] In contrast, electron localization has been recently predicted to be energetically favorable in many tin halide perovskites, with bipolaronic states as the most stable form of self-trapped electrons.[42]

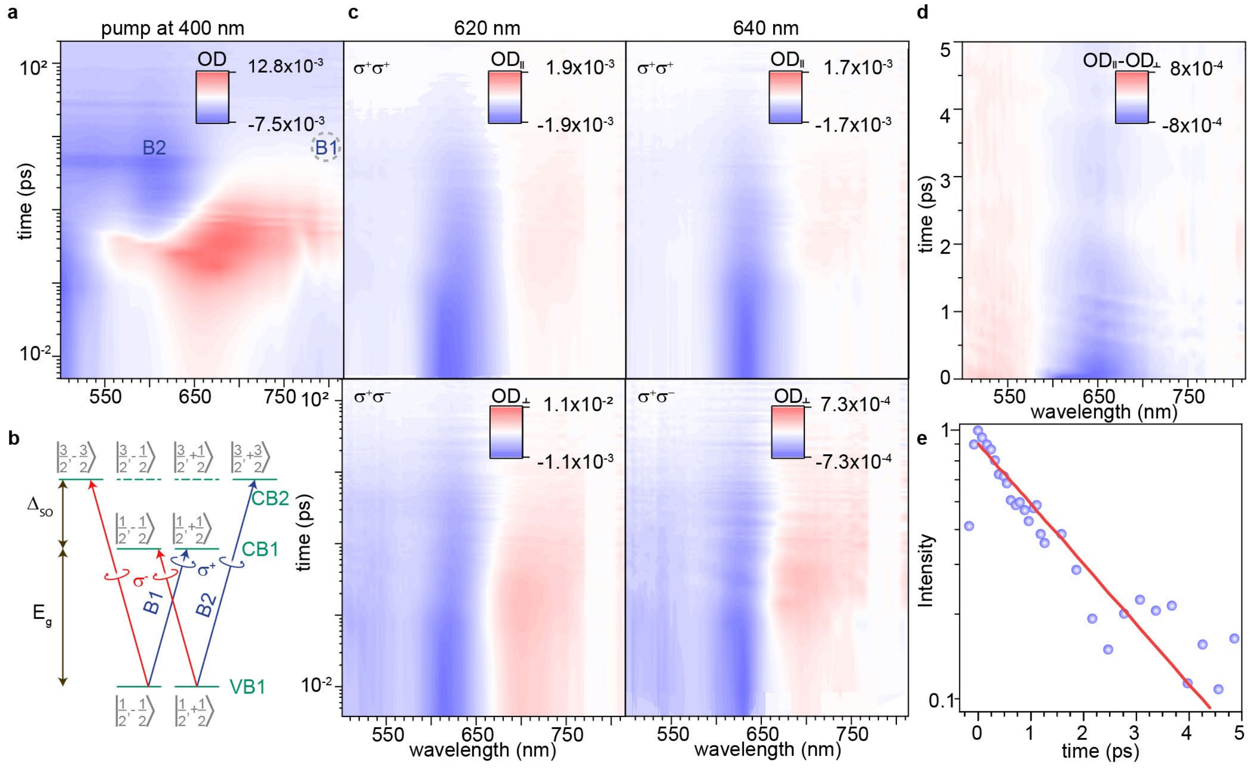

Figure SN4F1. Pump-probe TA spectroscopy of colloidal $FASnI_3$ NCs. (a) pseudo-color 2D plot for room temperature TA pumped at 400 nm. (b) Optical selection rules in $FASnI_3$; the spin-polarized states are labeled using $|J, m_J\rangle$. The solid and dashed lines of the J = 3/2 states represent heavy- (HE) and light (LE) electrons correspondingly. (c) Pseudo-color 2D plots for room temperature TA pumped at 620 nm (left panel) and 640 nm (right panel) with circularly co- (top) and cross-polarized (bottom) pump/probe photons. (d) The net spin pseudo-color 2D plot obtained as a difference between co-and cross-polarized TA spectra pumped at 620 nm. (e) Experimental (blue dots) net spin kinetics with 650 nm probe photons and single-exponent fit (red line).

12. Supplementary note 5: doping $FASnI_3$ NCs with small A-site or bifunctional cations

We test several cations which are expected to have a different effect on the lattice, namely:

(i) ethylenediammonium (EDA) and 2-hydroxyethylammonium (HEA) known to form so-called "hollow" structures with many halide perovskites;[1, 43-47] doping with these cations induces the mild expansion of the lattice and increases the degree of disorder in the structure

(ii) Cs cation, which is smaller than FA and is expected to induce the lattice contraction; doping bulk tin halide perovskites with Cs is known to improve the lattice symmetry[48-49]

Despite the difference in the actual distortion that these cations introduce to the perovskite lattice, all of them do not alter the structure dimensionality, and all of them have been reported to enhance the stability of tin iodide perovskites.

All three cations allow for synthesizing colloidally stable functionalized $FASnI_3$ NCs.

Introduced distortions increase the bandgap of NCs by as much as 0.45 eV for HEA (Figure 5a of the main text).

13. Supplementary Figures

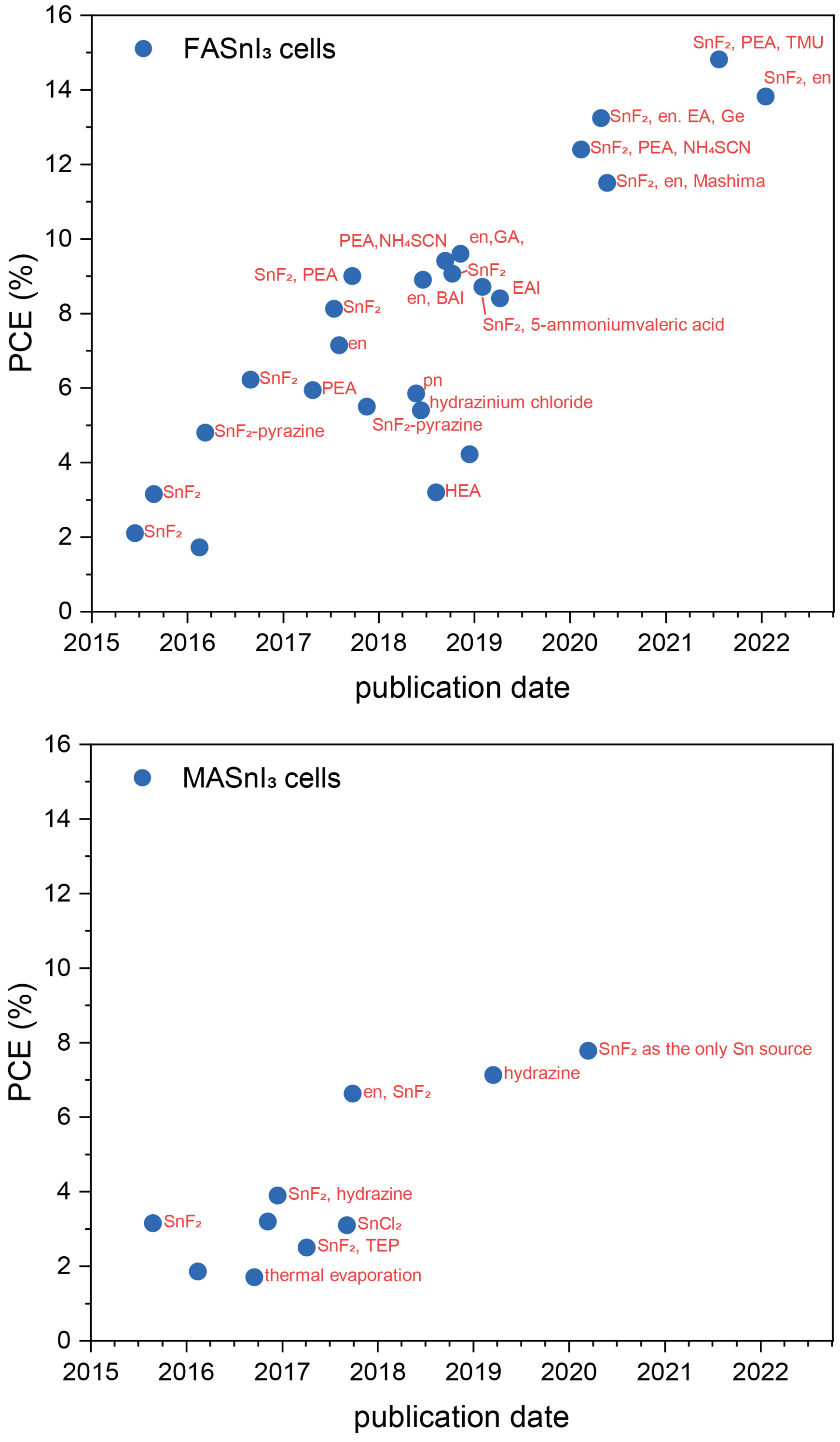

Figure S1. Evolution of the PCE of $FASnI_3$ and $MASnI_3$ solar cells.

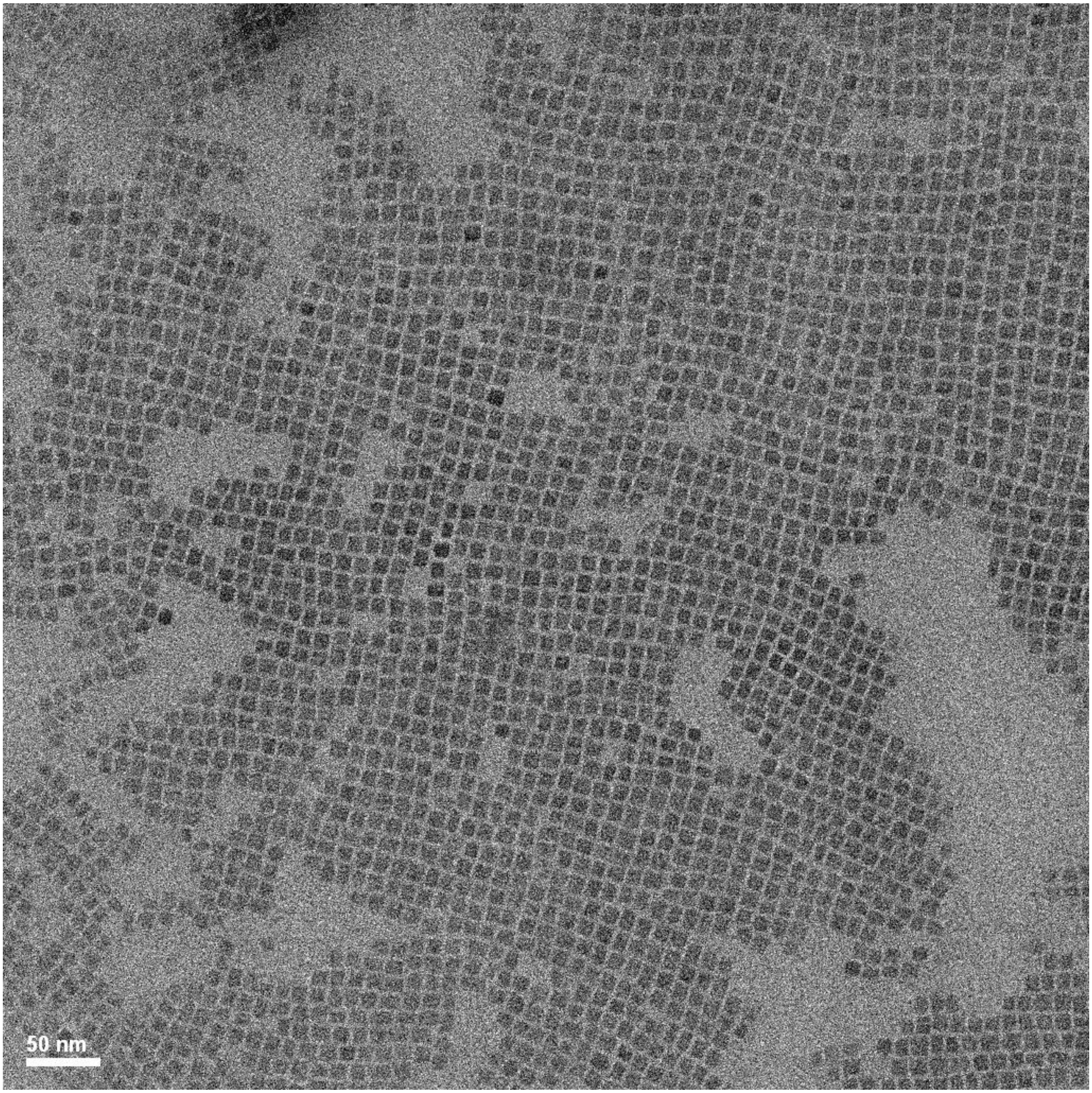

Figure S2. TEM of $FASnI_3$ NCs synthesized in ODE.

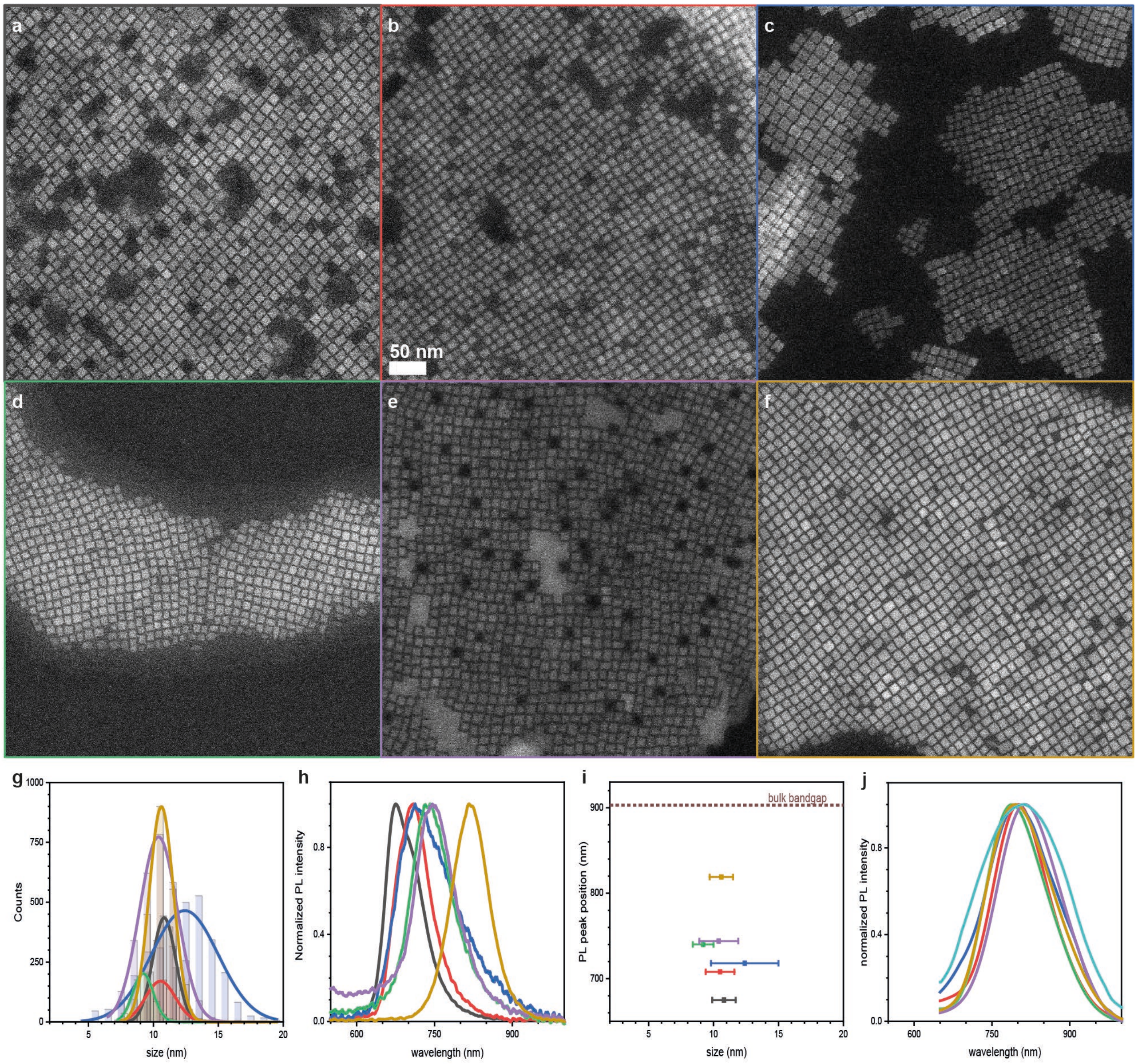

Figure S3. Poor correlation of the PL spectra and NCs size for $FASnI_3$ NCs synthesized in ODE. (a)-(f) TEM of several batches of $FASnI_3$ NCs synthesized in ODE. The scale bar in (b) is for all images. (g) Corresponding size histograms. (h) PL spectra of the samples from (a)-(f). (i) shows PL peak maxima in respect to the size of NCs indicating strong variation of the PL spectrum even for NCs of similar size. (j) Variation of the PL spectra for the samples synthesized in fully optimized conditions.

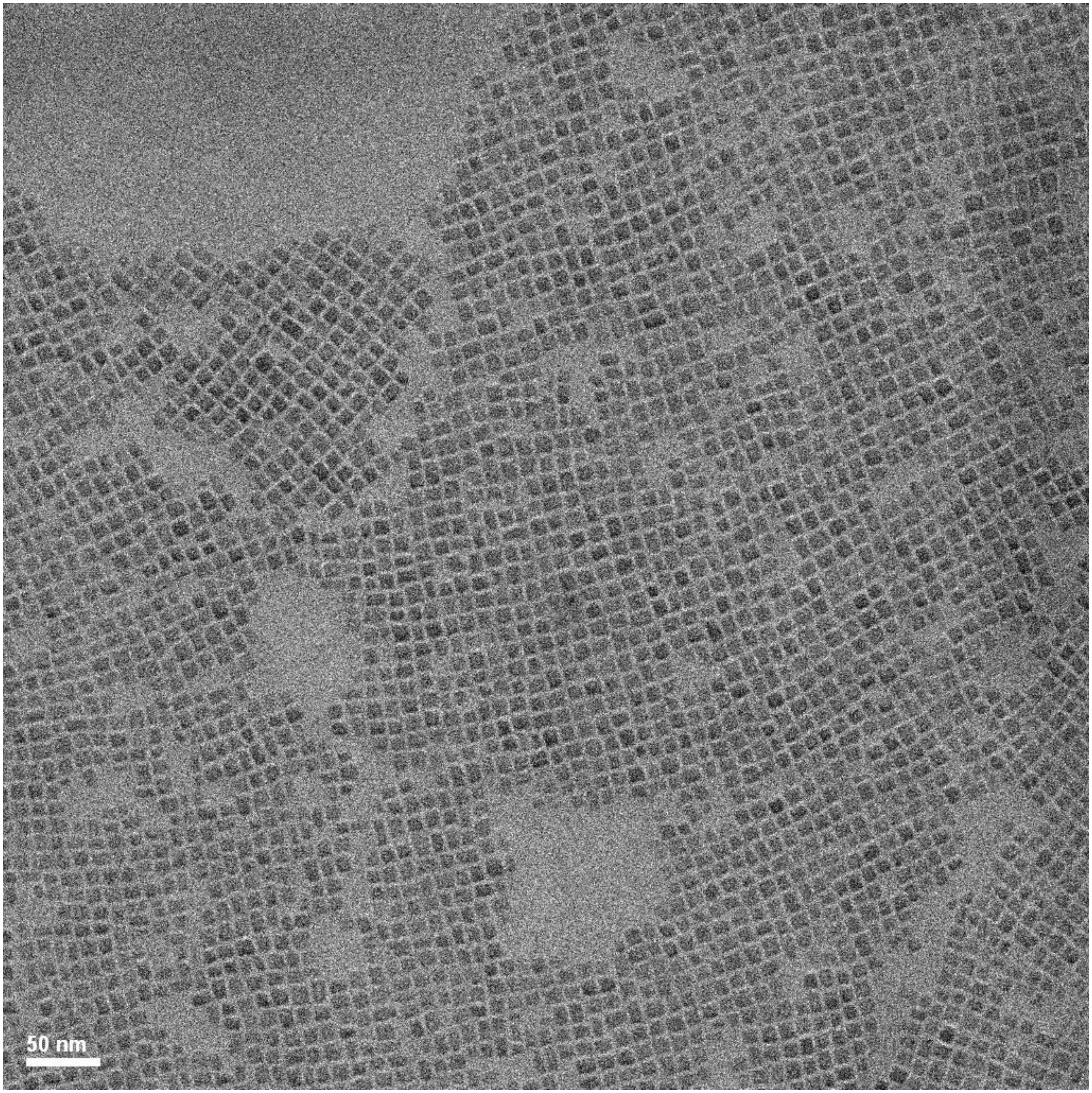

Figure S4. TEM of $FASnI_3$ NCs synthesized in mesitylene.

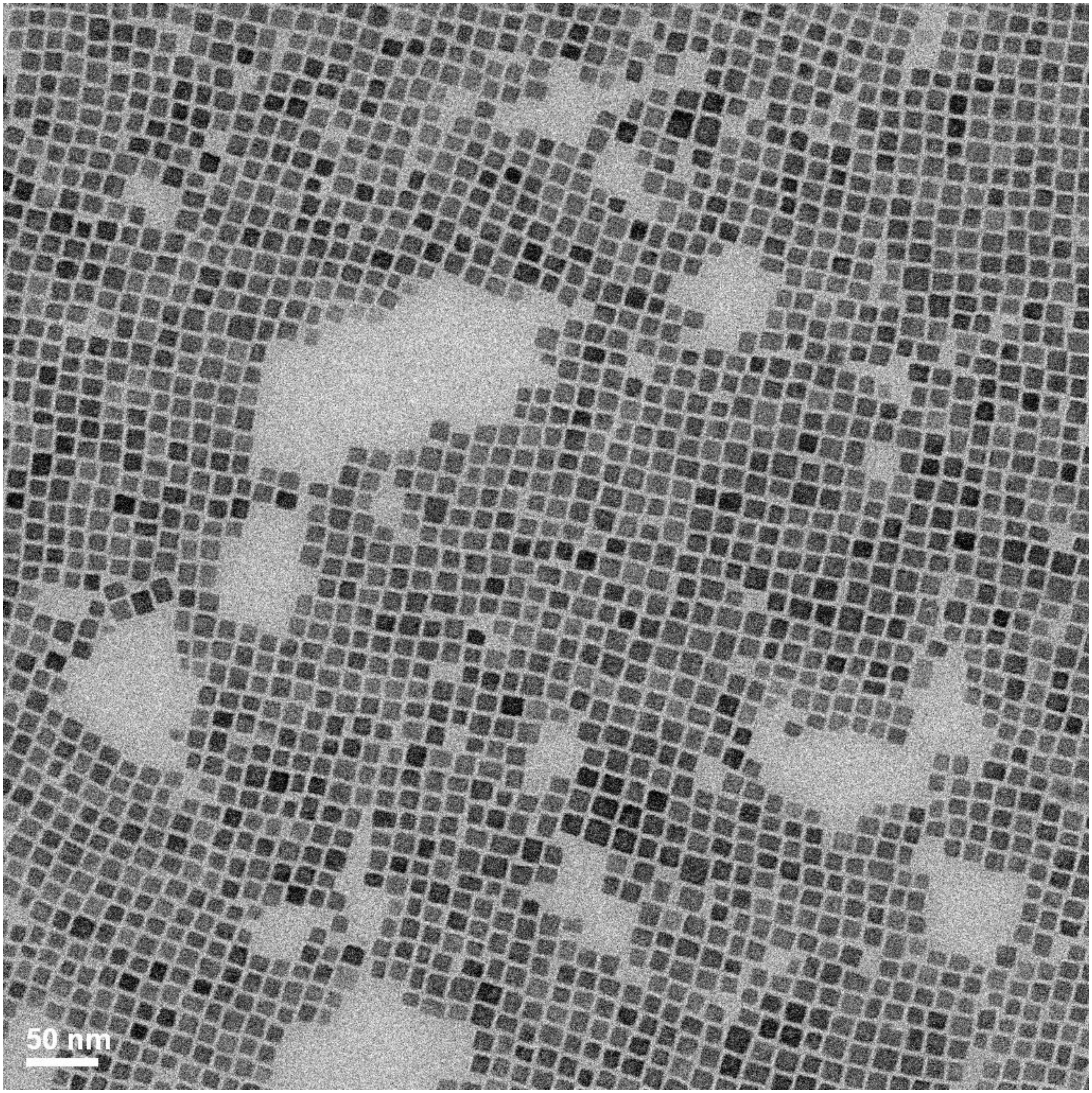

Figure S5. TEM of $FASnI_3$ NCs synthesized in toluene.

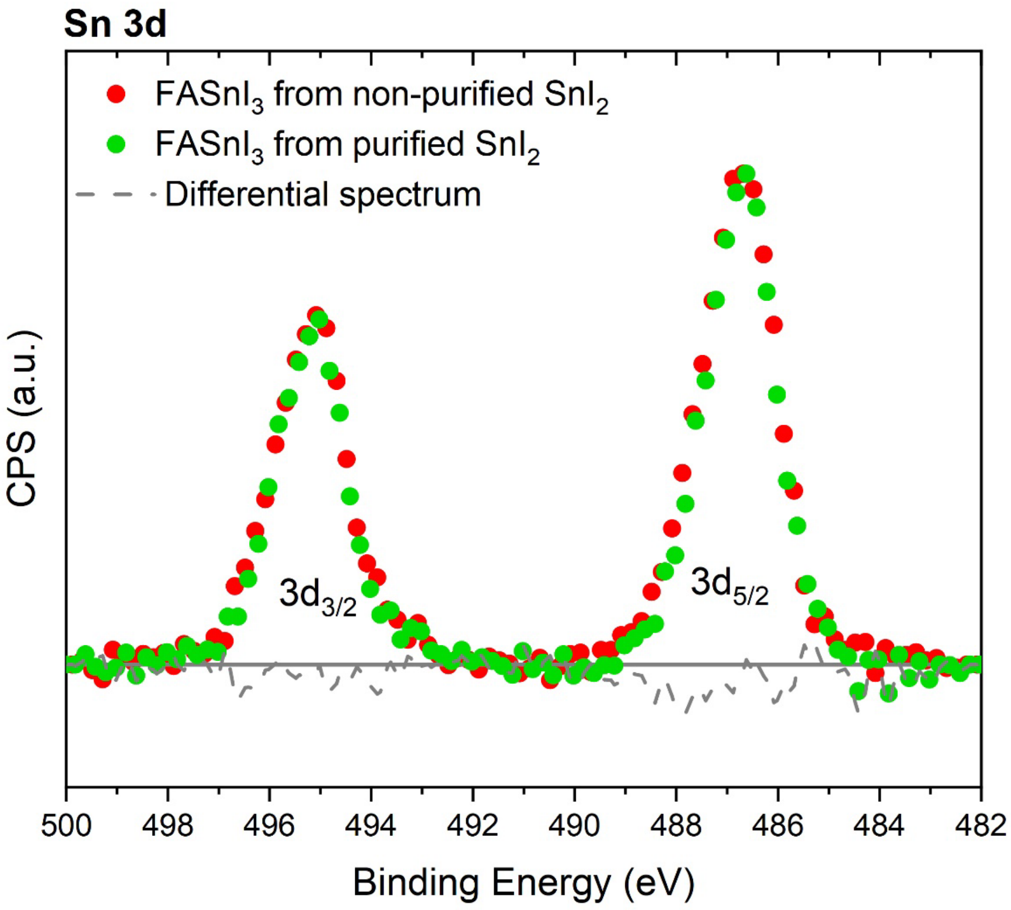

Figure S6. XPS Sn 3d spectra for $FASnI_3$ NC films made from non-purified (red dots) and purified (green dots) SnI_2 with corresponding differential spectrum showing a great similarity between the two.

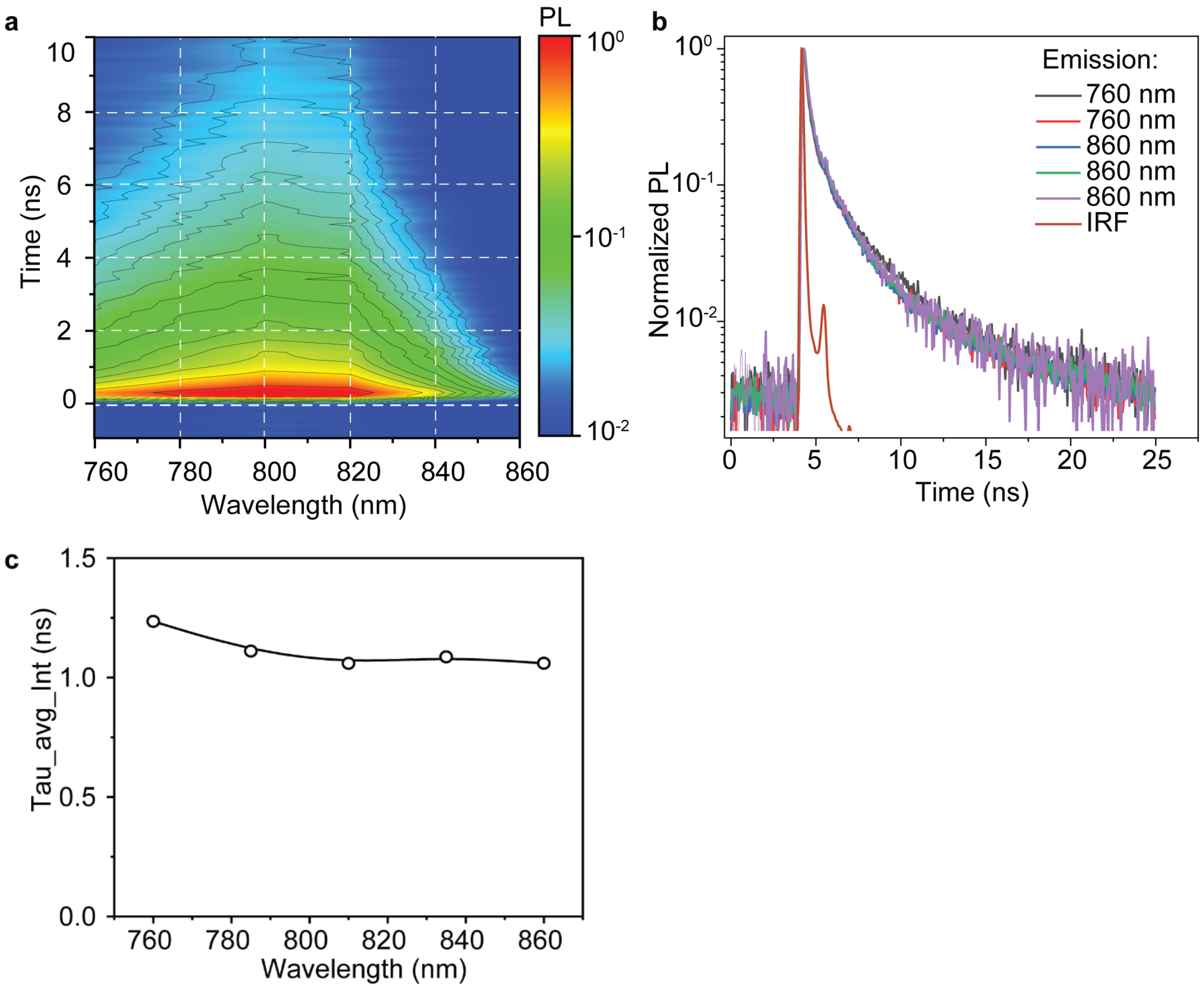

Figure S7. (a) Time-resolved emission spectrum (TRES) of colloidal $FASnI_3$ NCs. (b) TR PL at specified wavelengths plotted together with the instrument response function (IRF). (c) Intensity-averaged PL lifetime independence on wavelength of emission.

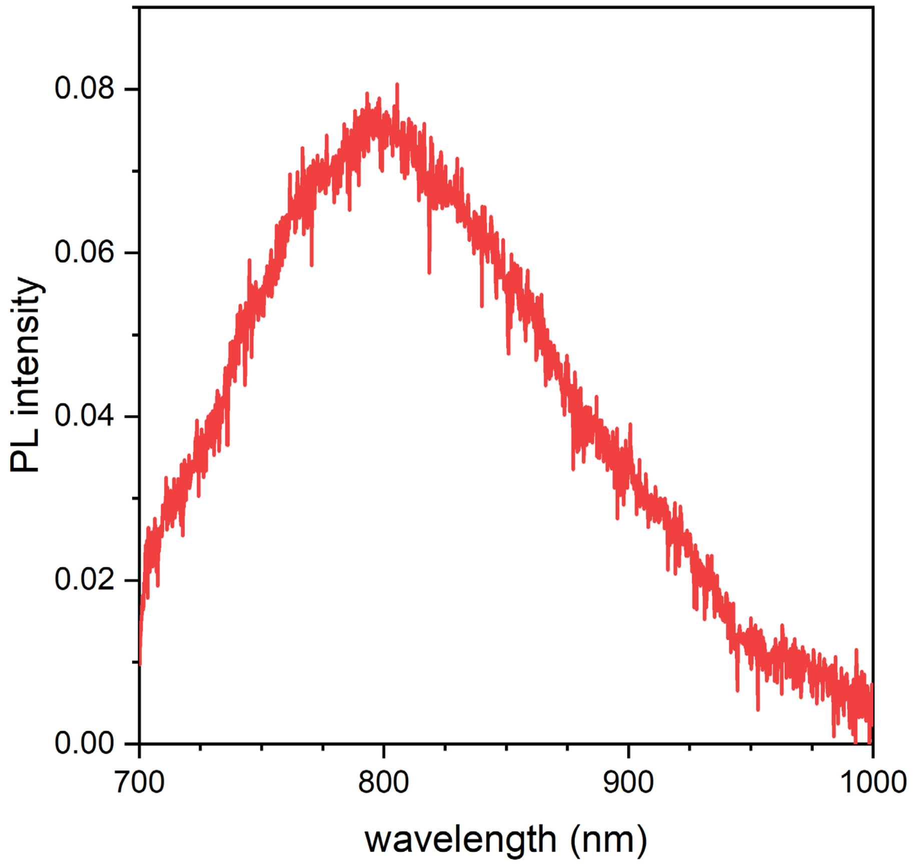

Figure S8. Photoluminescence spectrum of colloidal $FASnI_3$ NCs excited at 635 nm.

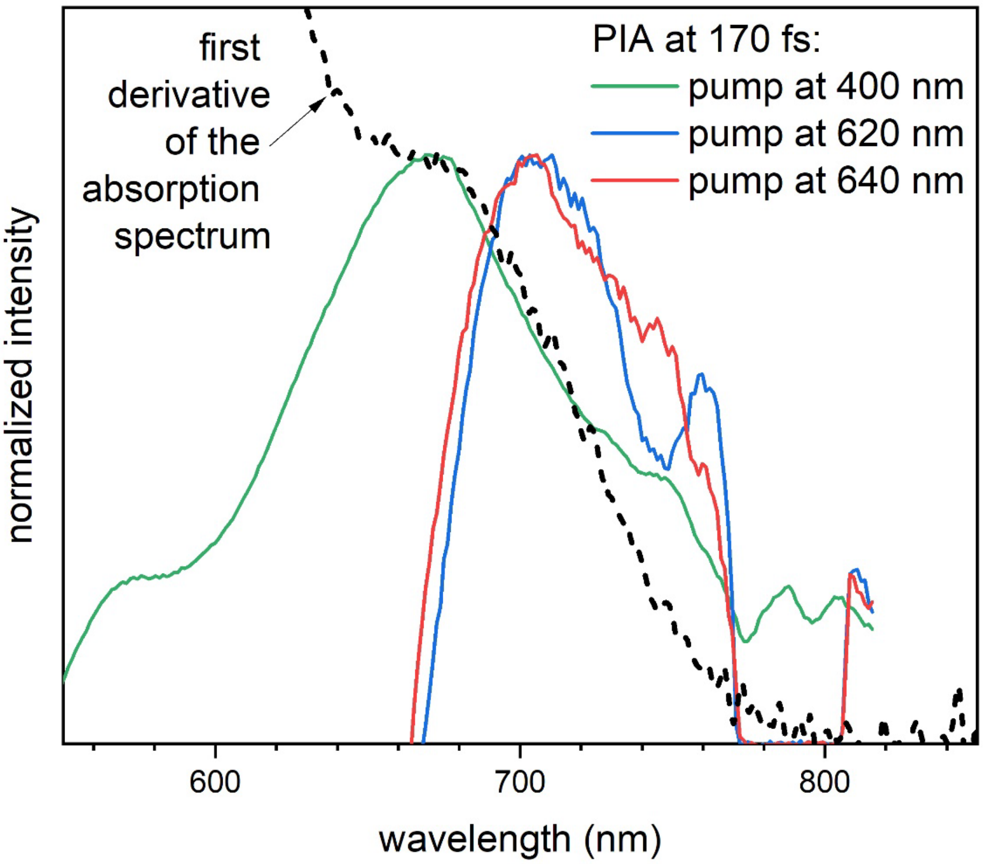

Figure S9. Comparison of the PIA of colloidal FASnI3 NCs for pumping at 400 nm (green) and in resonance with the bleach B2 (red and blue) indicates that observed PIA bands may have different origin. PIA observed when NCs are pumped in resonance is attributed to biexcitons. PIA observed when NCs are pumped well above the bandgap coincides with the first derivative of the absorption spectrum (dashed line), indicating that it may originate from altering the bandgap. This PIA is attributed to the deformation of the lattice due to hot carriers, which may create additional states but disappears with carriers cooling.

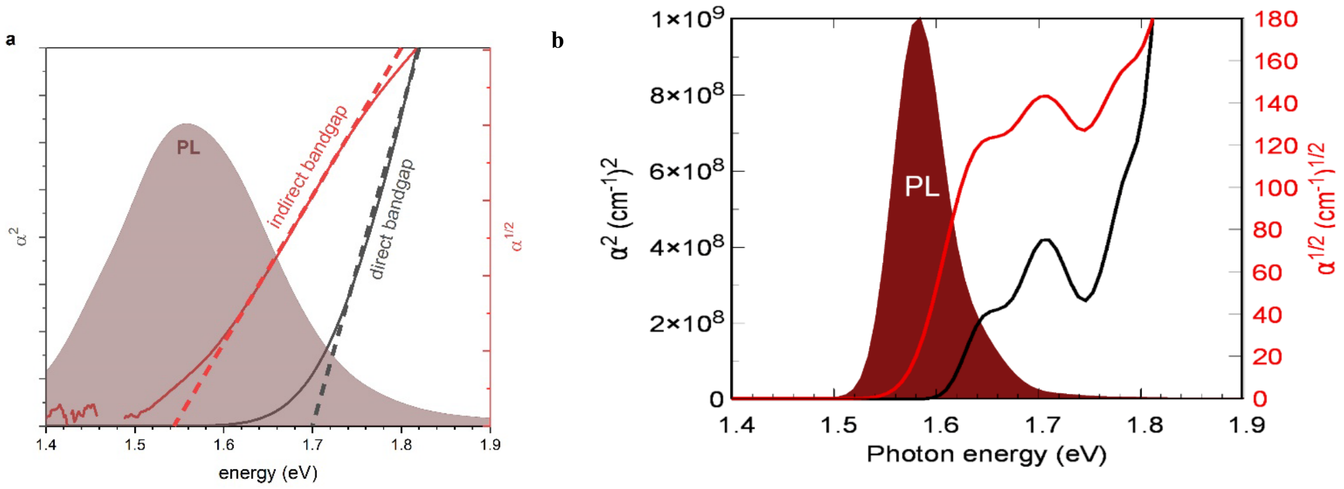

Figure S10. Experimental (a) and calculated (b) absorption spectrum of pure $FASnI_3$ NCs in Tauc plots for direct and indirect allowed transitions in comparison with the observed PL spectrum.

14. Supplementary references:

(1) Jokar, E.; Chien, C.-H.; Fathi, A.; Rameez, M.; Chang, Y.-H.; Diau, E. W.-G. Slow surface passivation and crystal relaxation with additives to improve device performance and durability for tin-based perovskite solar cells. *Energy & Environmental Science* **2018,** *11* (9), 2353-2362.

(2) Willmott, P. R.; Meister, D.; Leake, S. J.; Lange, M.; Bergamaschi, A.; Boge, M.; Calvi, M.; Cancellieri, C.; Casati, N.; Cervellino, A.; Chen, Q.; David, C.; Flechsig, U.; Gozzo, F.; Henrich, B.; Jaggi-Spielmann, S.; Jakob, B.; Kalichava, I.; Karvinen, P.; Krempasky, J.; Ludeke, A.; Luscher, R.; Maag, S.; Quitmann, C.; Reinle-Schmitt, M. L.; Schmidt, T.; Schmitt, B.; Streun, A.; Vartiainen, I.; Vitins, M.; Wang, X.; Wullschleger, R. The Materials Science beamline upgrade at the Swiss Light Source. *Journal of Synchrotron Radiation* **2013,** *20* (5), 667-682.

(3) Bergamaschi, A.; Cervellino, A.; Dinapoli, R.; Gozzo, F.; Henrich, B.; Johnson, I.; Kraft, P.; Mozzanica, A.; Schmitt, B.; Shi, X. The MYTHEN detector for X-ray powder diffraction experiments at the Swiss Light Source. *Journal of Synchrotron Radiation* **2010,** *17* (5), 653-668.

(4) Paalman, H. H.; Pings, C. J. Numerical Evaluation of X-Ray Absorption Factors for Cylindrical Samples and Annular Sample Cells. *Journal of Applied Physics* **1962,** *33* (8), 2635-2639.

(5) Badocco, D.; Di Marco, V.; Piovan, A.; Caniato, R.; Pastore, P. A procedure for the quantification of total iodine by inductively coupled plasma mass spectrometry, and its application to the determination of iodine in algae sampled in the lagoon of Venice. *Analytical Methods* **2016,** *8* (41), 7545-7551.

(6) Tinggi, U.; Schoendorfer, N.; Davies, P. S. W.; Scheelings, P.; Olszowy, H. Determination of iodine in selected foods and diets by inductively coupled plasma-mass spectrometry. *Pure and Applied Chemistry* **2011,** *84* (2), 291-299.

(7) Coelho, A. TOPAS and TOPAS-Academic: an optimization program integrating computer algebra and crystallographic objects written in C++. *Journal of Applied Crystallography* **2018,** *51* (1), 210-218.

(8) Cervellino, A.; Frison, R.; Bertolotti, F.; Guagliardi, A. DEBUSSY 2.0: the new release of a Debye user system for nanocrystalline and/or disordered materials. *Journal of Applied Crystallography* **2015,** *48* (6), 2026-2032.

(9) Weller, M. T.; Weber, O. J.; Frost, J. M.; Walsh, A. Cubic Perovskite Structure of Black Formamidinium Lead Iodide, α-$[HC(NH_2)_2]PbI_3$, at 298 K. *The Journal of Physical Chemistry Letters* **2015,** *6* (16), 3209-3212.

(10) Debye, P. Zerstreuung von Röntgenstrahlen. *Annalen der Physik* **1915,** *351* (6), 809-823.

(11) Kahmann, S.; Nazarenko, O.; Shao, S.; Hordiichuk, O.; Kepenekian, M.; Even, J.; Kovalenko, M. V.; Blake, G. R.; Loi, M. A. Negative Thermal Quenching in $FASnI_3$ Perovskite Single Crystals and Thin Films. *ACS Energy Letters* **2020,** *5* (8), 2512-2519.

(12) Qiu, X.; Thompson, J. W.; Billinge, S. J. L. PDFgetX2: a GUI-driven program to obtain the pair distribution function from X-ray powder diffraction data. *Journal of Applied Crystallography* **2004,** *37* (4), 678.

(13) Jensen, K. M. Ø.; Christensen, M.; Juhas, P.; Tyrsted, C.; Bøjesen, E. D.; Lock, N.; Billinge, S. J. L.; Iversen, B. B. Revealing the Mechanisms behind SnO_2 Nanoparticle Formation and Growth during Hydrothermal Synthesis: An In Situ Total Scattering Study. *Journal of the American Chemical Society* **2012,** *134* (15), 6785-6792.

(14) Shinotsuka, H.; Tanuma, S.; Powell, C. J.; Penn, D. R. Calculations of electron inelastic mean free paths. X. Data for 41 elemental solids over the 50 eV to 200 keV range with the relativistic full Penn algorithm. *Surface and Interface Analysis* **2015,** *47* (9), 871-888.

(15) Giannozzi, P.; Baroni, S.; Bonini, N.; Calandra, M.; Car, R.; Cavazzoni, C.; Ceresoli, D.; Chiarotti, G. L.; Cococcioni, M.; Dabo, I.; Dal Corso, A.; de Gironcoli, S.; Fabris, S.; Fratesi, G.; Gebauer, R.; Gerstmann, U.; Gougoussis, C.; Kokalj, A.; Lazzeri, M.; Martin-Samos, L.; Marzari, N.; Mauri, F.; Mazzarello, R.; Paolini, S.; Pasquarello, A.; Paulatto, L.; Sbraccia, C.; Scandolo, S.; Sclauzero, G.; Seitsonen, A. P.; Smogunov, A.; Umari, P.; Wentzcovitch, R. M. QUANTUM ESPRESSO: a modular and open-source software project for quantum simulations of materials. *Journal of Physics: Condensed Matter* **2009,** *21* (39), 395502.

(16) Giannozzi, P.; Andreussi, O.; Brumme, T.; Bunau, O.; Buongiorno Nardelli, M.; Calandra, M.; Car, R.; Cavazzoni, C.; Ceresoli, D.; Cococcioni, M.; Colonna, N.; Carnimeo, I.; Dal Corso, A.; de Gironcoli, S.; Delugas, P.; DiStasio, R. A.; Ferretti, A.; Floris, A.; Fratesi, G.; Fugallo, G.; Gebauer, R.; Gerstmann, U.; Giustino, F.; Gorni, T.; Jia, J.; Kawamura, M.; Ko, H. Y.; Kokalj, A.; Küçükbenli, E.; Lazzeri, M.; Marsili, M.; Marzari, N.; Mauri, F.; Nguyen, N. L.; Nguyen, H. V.; Otero-de-la-Roza, A.; Paulatto, L.; Poncé, S.; Rocca, D.; Sabatini, R.; Santra, B.; Schlipf, M.; Seitsonen, A. P.; Smogunov, A.; Timrov, I.; Thonhauser, T.; Umari, P.; Vast, N.; Wu, X.; Baroni, S. Advanced capabilities for materials modelling with Quantum ESPRESSO. *Journal of Physics: Condensed Matter* **2017,** *29* (46), 465901.

(17) Perdew, J. P.; Ruzsinszky, A.; Csonka, G. I.; Vydrov, O. A.; Scuseria, G. E.; Constantin, L. A.; Zhou, X.; Burke, K. Restoring the Density-Gradient Expansion for Exchange in Solids and Surfaces. *Physical Review Letters* **2008,** *100* (13), 136406.

(18) Hamann, D. R. Optimized norm-conserving Vanderbilt pseudopotentials. *Physical Review B* **2013,** *88* (8), 085117.

(19) Zhao, X.-G.; Dalpian, G. M.; Wang, Z.; Zunger, A. Polymorphous nature of cubic halide perovskites. *Physical Review B* **2020,** *101* (15), 155137.

(20) Baroni, S.; de Gironcoli, S.; Dal Corso, A.; Giannozzi, P. Phonons and related crystal properties from density-functional perturbation theory. *Reviews of Modern Physics* **2001,** *73* (2), 515-562.

(21) Zacharias, M.; Giustino, F. Theory of the special displacement method for electronic structure calculations at finite temperature. *Physical Review Research* **2020,** *2* (1), 013357.

(22) Popescu, V.; Zunger, A. Extracting E versus k effective band structure from supercell calculations on alloys and impurities. *Physical Review B* **2012,** *85* (8), 085201.

(23) Poncé, S.; Margine, E. R.; Verdi, C.; Giustino, F. EPW: Electron–phonon coupling, transport and superconducting properties using maximally localized Wannier functions. *Computer Physics Communications* **2016,** *209*, 116-133.

(24) Zacharias, M.; Giustino, F. One-shot calculation of temperature-dependent optical spectra and phonon-induced band-gap renormalization. *Physical Review B* **2016,** *94* (7), 075125.

(25) van Roosbroeck, W.; Shockley, W. Photon-Radiative Recombination of Electrons and Holes in Germanium. *Physical Review* **1954,** *94* (6), 1558-1560.

(26) Wong, A. B.; Bekenstein, Y.; Kang, J.; Kley, C. S.; Kim, D.; Gibson, N. A.; Zhang, D.; Yu, Y.; Leone, S. R.; Wang, L.-W.; Alivisatos, A. P.; Yang, P. Strongly Quantum Confined Colloidal Cesium Tin Iodide Perovskite Nanoplates: Lessons for Reducing Defect Density and Improving Stability. *Nano Letters* **2018,** *18* (3), 2060-2066.

(27) Jellicoe, T. C.; Richter, J. M.; Glass, H. F. J.; Tabachnyk, M.; Brady, R.; Dutton, S. E.; Rao, A.; Friend, R. H.; Credgington, D.; Greenham, N. C.; Böhm, M. L. Synthesis and Optical Properties of Lead-Free Cesium Tin Halide Perovskite Nanocrystals. *Journal of the American Chemical Society* **2016,** *138* (9), 2941-2944.

(28) Wieczorek, A. L., H.; Pious, J.; Fu, F.; Siol, S. Resolving oxidation states and Sn-halide interactions of perovskites through Auger parameter analysis in XPS. *Adv. Mater. Interfaces* **2022,** *2201828.*

(29) Bandara, R. M. I.; Jayawardena, K. D. G. I.; Adeyemo, S. O.; Hinder, S. J.; Smith, J. A.; Thirimanne, H. M.; Wong, N. C.; Amin, F. M.; Freestone, B. G.; Parnell, A. J.; Lidzey, D. G.; Joyce, H. J.; Sporea, R. A.; Silva, S. R. P. Tin(iv) dopant removal through anti-solvent engineering enabling tin based perovskite solar cells with high charge carrier mobilities. *Journal of Materials Chemistry C* **2019,** *7* (27), 8389-8397.

(30) Naumkin, A. V. K.-V., A.; Gaarenstroom, S. W.; Powell, C. J. NIST X-ray Photoelectron Spectroscopy Database. Version 4.1. Date of access: 03.02.2023. *http://srdata.nist.gov/xps/*.

(31) Mulder, J. T.; du Fossé, I.; Alimoradi Jazi, M.; Manna, L.; Houtepen, A. J. Electrochemical p-Doping of $CsPbBr_3$ Perovskite Nanocrystals. *ACS Energy Letters* **2021,** *6* (7), 2519-2525.

(32) Nakamura, T.; Yakumaru, S.; Truong, M. A.; Kim, K.; Liu, J.; Hu, S.; Otsuka, K.; Hashimoto, R.; Murdey, R.; Sasamori, T.; Kim, H. D.; Ohkita, H.; Handa, T.; Kanemitsu, Y.; Wakamiya, A. Sn(IV)-free tin perovskite films realized by in situ Sn(0) nanoparticle treatment of the precursor solution. *Nature Communications* **2020,** *11* (1), 3008.

(33) Maes, J.; Balcaen, L.; Drijvers, E.; Zhao, Q.; De Roo, J.; Vantomme, A.; Vanhaecke, F.; Geiregat, P.; Hens, Z. Light Absorption Coefficient of $CsPbBr_3$ Perovskite Nanocrystals. *The Journal of Physical Chemistry Letters* **2018,** *9* (11), 3093-3097.

(34) Ghimire, K.; Zhao, D.; Yan, Y.; Podraza, N. J. Optical response of mixed methylammonium lead iodide and formamidinium tin iodide perovskite thin films. *AIP Advances* **2017,** *7* (7), 075108.

(35) Savill, K. J.; Klug, M. T.; Milot, R. L.; Snaith, H. J.; Herz, L. M. Charge-Carrier Cooling and Polarization Memory Loss in Formamidinium Tin Triiodide. *The Journal of Physical Chemistry Letters* **2019,** *10* (20), 6038-6047.

(36) Li, Y.; Luo, X.; Liu, Y.; Lu, X.; Wu, K. Size- and Composition-Dependent Exciton Spin Relaxation in Lead Halide Perovskite Quantum Dots. *ACS Energy Letters* **2020,** *5* (5), 1701-1708.

(37) Liang, W.; Li, Y.; Xiang, D.; Han, Y.; Jiang, Q.; Zhang, W.; Wu, K. Efficient Optical Orientation and Slow Spin Relaxation in Lead-Free $CsSnBr_3$ Perovskite Nanocrystals. *ACS Energy Letters* **2021,** *6* (5), 1670-1676.

(38) Huang, L.-y.; Lambrecht, W. R. L. Electronic band structure, phonons, and exciton binding energies of halide perovskites $CsSnCl_3$, $CsSnBr_3$, and $CsSnI_3$. *Physical Review B* **2013,** *88* (16), 165203.

(39) Peng, L.; Xie, W. Theoretical and experimental investigations on the bulk photovoltaic effect in lead-free perovskites $MASnI_3$ and $FASnI_3$. *RSC Advances* **2020,** *10* (25), 14679-14688.

(40) Siol, S.; Mann, J.; Newman, J.; Miyayama, T.; Watanabe, K.; Schmutz, P.; Cancellieri, C.; Jeurgens, L. P. H. Concepts for chemical state analysis at constant probing depth by lab-based XPS/HAXPES combining soft and hard X-ray sources. *Surface and Interface Analysis* **2020,** *52* (12), 802-810.

(41) Geiregat, P.; Rodá, C.; Tanghe, I.; Singh, S.; Di Giacomo, A.; Lebrun, D.; Grimaldi, G.; Maes, J.; Van Thourhout, D.; Moreels, I.; Houtepen, A. J.; Hens, Z. Localization-limited exciton oscillator strength in colloidal CdSe nanoplatelets revealed by the optically induced stark effect. *Light: Science & Applications* **2021,** *10* (1), 112.

(42) Ouhbi, H.; Ambrosio, F.; De Angelis, F.; Wiktor, J. Strong Electron Localization in Tin Halide Perovskites. *The Journal of Physical Chemistry Letters* **2021,** *12* (22), 5339-5343.

(43) Ke, W.; Stoumpos Constantinos, C.; Zhu, M.; Mao, L.; Spanopoulos, I.; Liu, J.; Kontsevoi Oleg, Y.; Chen, M.; Sarma, D.; Zhang, Y.; Wasielewski Michael, R.; Kanatzidis Mercouri, G. Enhanced photovoltaic performance and stability with a new type of hollow 3D perovskite {en}$FASnI_3$. *Science Advances* **2017,** *3* (8), e1701293.

(44) Ke, W.; Stoumpos, C. C.; Spanopoulos, I.; Chen, M.; Wasielewski, M. R.; Kanatzidis, M. G. Diammonium Cations in the $FASnI_3$ Perovskite Structure Lead to Lower Dark Currents and More Efficient Solar Cells. *ACS Energy Letters* **2018,** *3* (7), 1470-1476.

(45) Spanopoulos, I.; Ke, W.; Stoumpos, C. C.; Schueller, E. C.; Kontsevoi, O. Y.; Seshadri, R.; Kanatzidis, M. G. Unraveling the Chemical Nature of the 3D "Hollow" Hybrid Halide Perovskites. *Journal of the American Chemical Society* **2018,** *140* (17), 5728-5742.

(46) Tsai, C.-M.; Lin, Y.-P.; Pola, M. K.; Narra, S.; Jokar, E.; Yang, Y.-W.; Diau, E. W.-G. Control of Crystal Structures and Optical Properties with Hybrid Formamidinium and 2-Hydroxyethylammonium Cations for Mesoscopic Carbon-Electrode Tin-Based Perovskite Solar Cells. *ACS Energy Letters* **2018,** *3* (9), 2077-2085.

(47) Jokar, E.; Chien, C.-H.; Tsai, C.-M.; Fathi, A.; Diau, E. W.-G. Robust Tin-Based Perovskite Solar Cells with Hybrid Organic Cations to Attain Efficiency Approaching 10%. *Advanced Materials* **2019,** *31* (2), 1804835.

(48) Gao, W.; Chen, C.; Ran, C.; Zheng, H.; Dong, H.; Xia, Y.; Chen, Y.; Huang, W. A-Site Cation Engineering of Metal Halide Perovskites: Version 3.0 of Efficient Tin-Based Lead-Free Perovskite Solar Cells. *Advanced Functional Materials* **2020,** *30* (34), 2000794.

(49) Gao, W.; Ran, C.; Li, J.; Dong, H.; Jiao, B.; Zhang, L.; Lan, X.; Hou, X.; Wu, Z. Robust Stability of Efficient Lead-Free Formamidinium Tin Iodide Perovskite Solar Cells Realized by Structural Regulation. *The Journal of Physical Chemistry Letters* **2018,** *9* (24), 6999-7006.
